# Supplementary material for: Sox9 regulates alternative splicing and pancreatic beta cell function
Source: Nat Commun. 2024 Jan 18;15:588. doi: 10.1038/s41467-023-44384-8 (PMC10796970; doi:10.1038/s41467-023-44384-8)

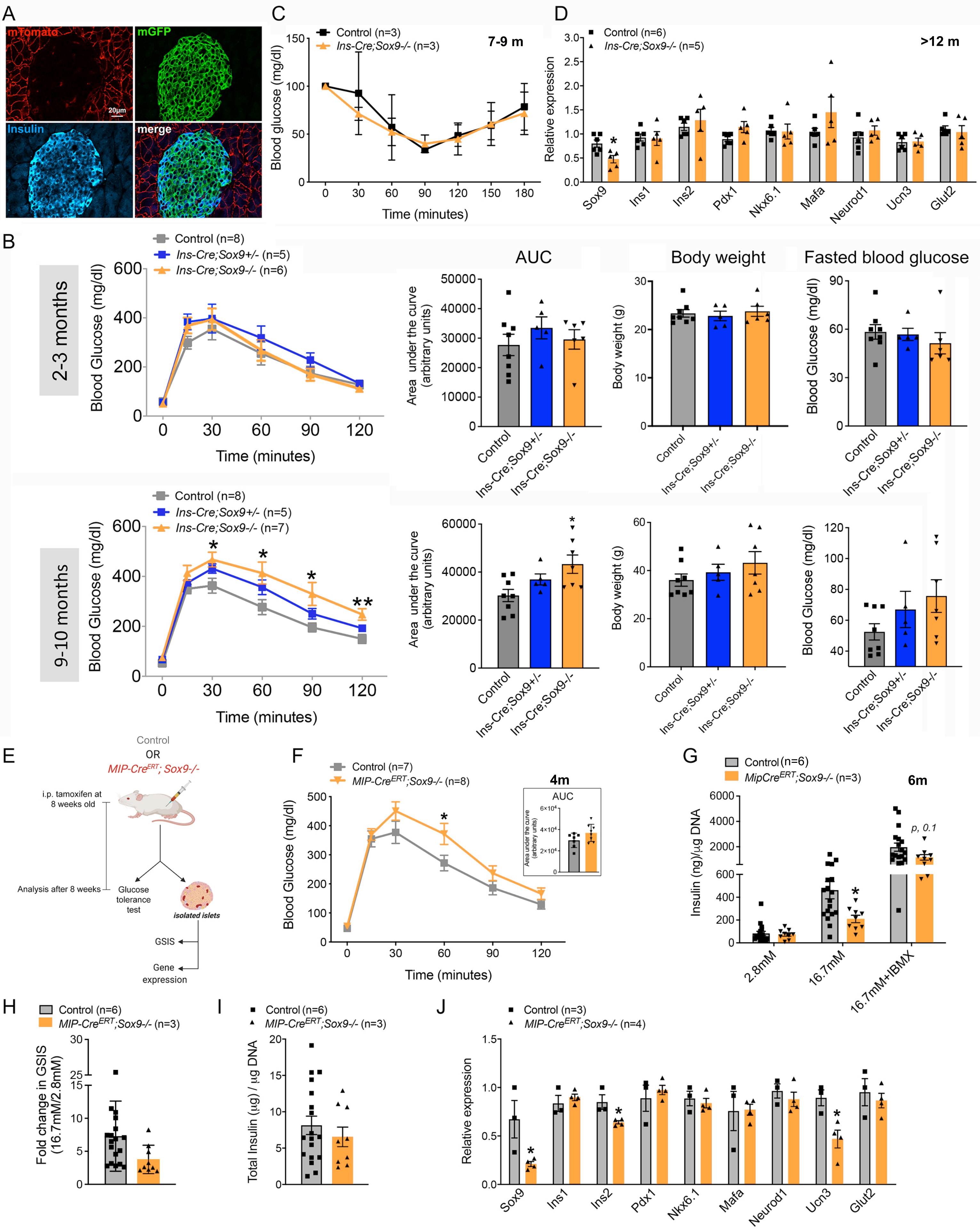

Figure S1. Progressive loss of beta cell function upon loss of Sox9. A. Recombination efficiency of Cre (*Ins-Cre*) using the mT/mG reporter mouse. Cre-expressing cells are GFP-positive (green), while unrecombined cells are Tomato-positive (red). Beta cells are marked by insulin staining (blue). Size bar, 20μm. **B.** Physiological measurements of control (grey) and Sox9 transgenic animals (heterozygous in blue, knockout in orange) over time. Glucose tolerance tests and the respective areas under the curves, body weight measurements, and fasted blood glucose are shown over distinct time points. N=8 control animals, 5 heterozygous animals, 6 knockout animals. *p*=0.028, 0.021, 0.013, and 0.004 at 30min, 60min, 90min, and 120min respectively. **C**. Insulin tolerance test carried out in control (black, n=3) and knockout (*Ins-Cre;Sox9-/-*, orange, n=3) animals at 7-9 months old. **D**. Quantitative PCR analysis on RNA from islets isolated from control (grey, n=6) or knockout (*Ins-Cre;Sox9-/-,* orange, n=5) animals (>12 months old). **E**. Strategy for deletion of Sox9 in adult beta cells using the *MIP-Cre^{ERT}* transgenic mouse. **F**. Glucose tolerance test after tamoxifen administration in control (grey, n=7) and knockout (*MIP-Cre^{ERT};Sox9-/-*, orange, n=8) animals. Inset shows area under the curve (AUC). *p*=0.049 at 60min. **G**. *In vitro* glucose-stimulated Insulin secretion from islets isolated from control (grey, n=6) or adult knockout animals (*MIP-Cre^{ERT};Sox9-/-*, orange, n=3). Stimulation index is shown in **H**. Single data points shown represent technical replicates. *p*=0.03 at high glucose. **I**. Total Insulin content in isolated islets from control (grey, n=6) or adult knockout (*MIP-Cre^{ERT};Sox9-/-*, orange, n=3) animals. Single data points shown represent technical replicates. **J**. Quantitative PCR analysis on RNA from islets isolated from control (grey, n=3) or adult knockout (*MIP-Cre^{ERT};Sox9-/-*, orange, n=4) animals. Error bars represent S.E.M. using a two-tailed Student's t test, *p<0.05, **p<0.005.

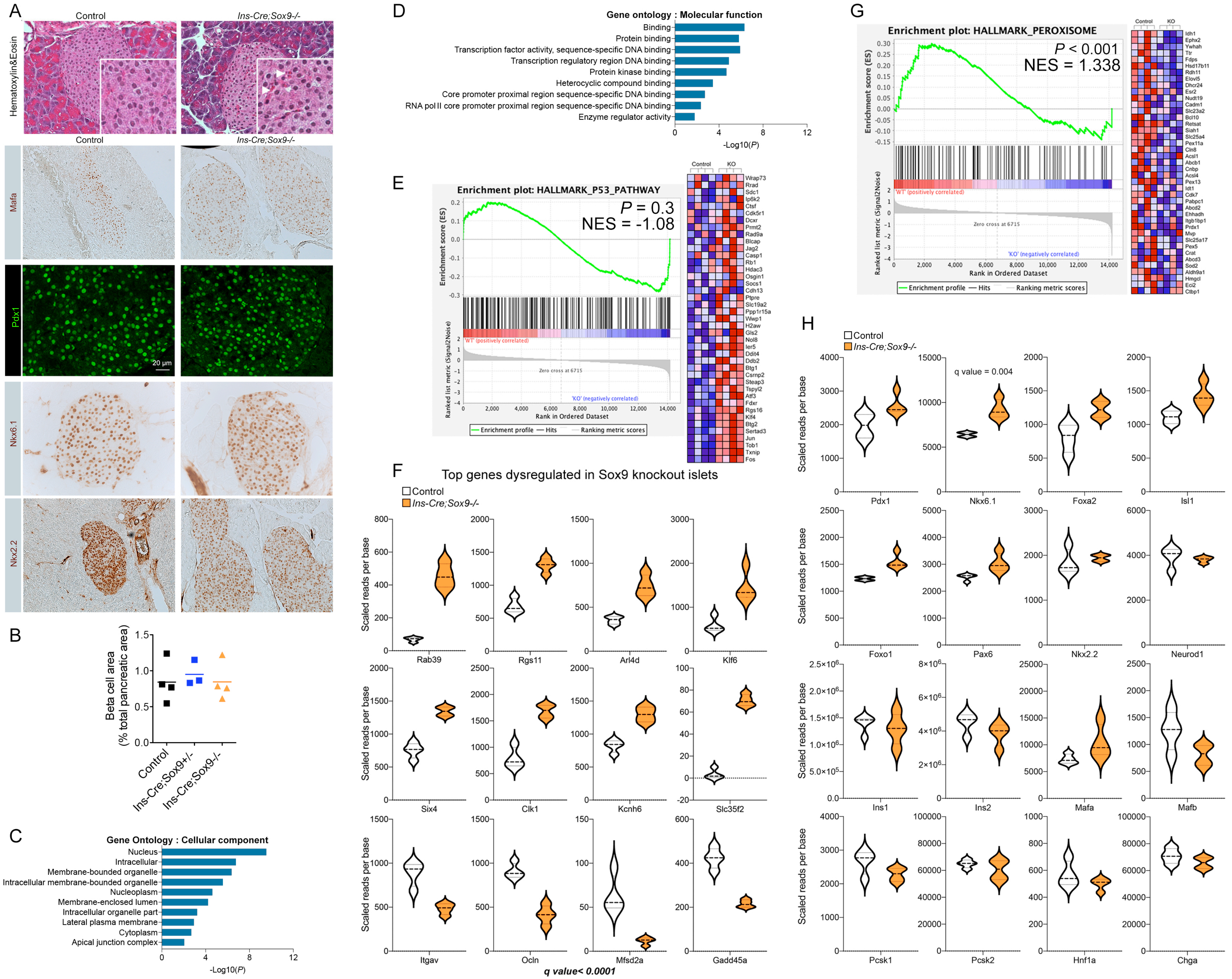

Figure S2. Pathways regulated by Sox9 in beta cells. A. Hematoxylin-Eosin staining of control and *Ins-Cre;Sox9-/-* islets. Immunohistochemical staining for Mafa, Nkx2.2 and Nkx6.1, and immunocytochemistry for Pdx1 are shown. **B.** Islet mass quantification for control, heterozygous (*Ins-Cre;Sox9+/-*), and homozygous knockouts (*Ins-Cre;Sox9-/-*) of Sox9. **C, D**. Gene ontology analysis (*Cellular Component* and *Molecular Function*) for the differential gene expression between control and knockout (*Ins-Cre;Sox9-/-*) islets. **E**. GSEA analysis of genes participating in the "**P53 Pathway**". Heat maps of the genes populating this pathway are shown. **F**. Top dysregulated genes depicted as violin plots (q value < 0.0001). **G**. "**Peroxisome**" pathway genes are downregulated in the knockout islets. **H**. Violin plots for gene counts of canonical beta cell markers in control (black) and *Ins-Cre;Sox9-/-* (orange) islets.

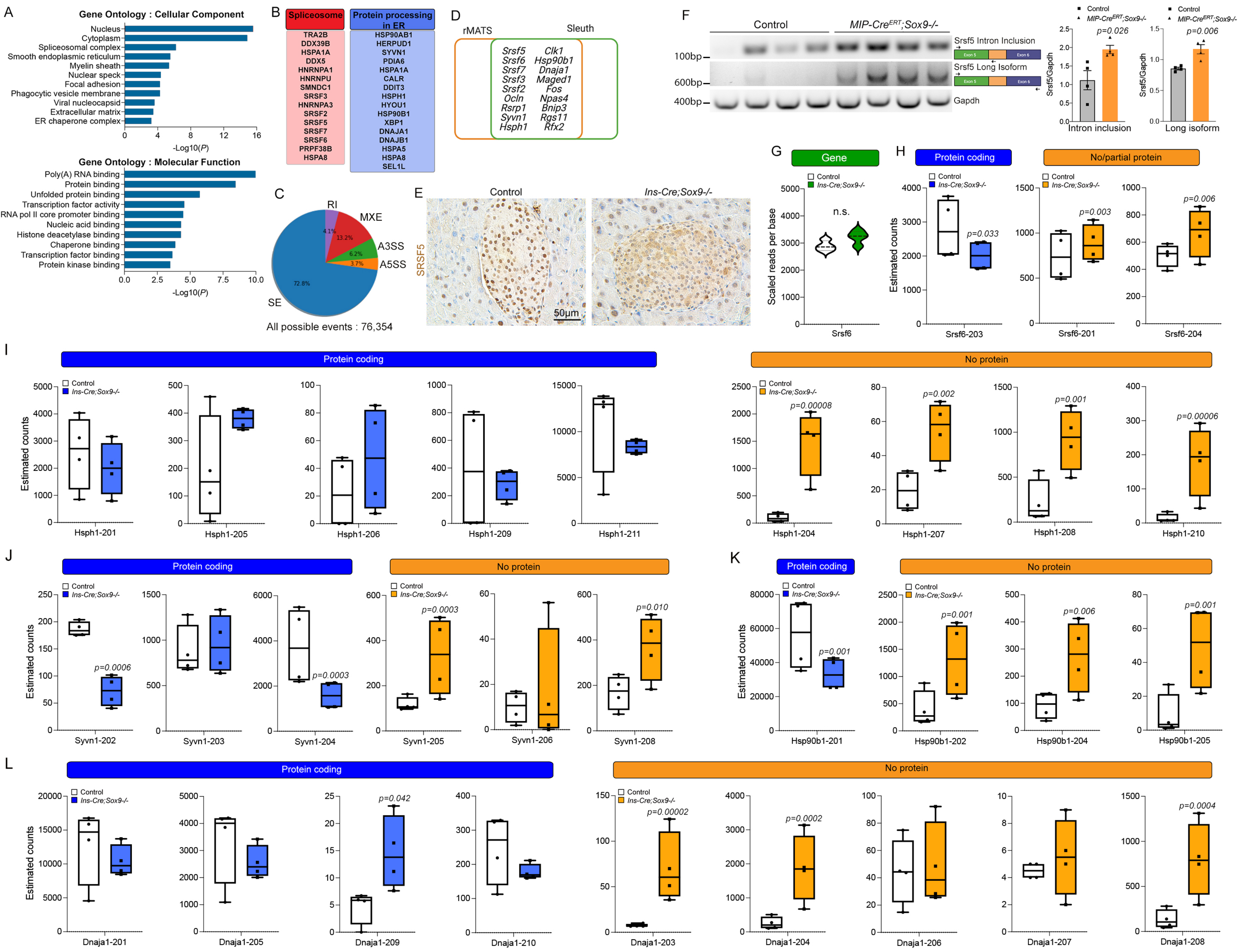

Figure S3. Change in alternative splicing upon loss of Sox9 in murine cells. A. Gene ontology analysis (*Cellular Component* and *Molecular Function*) of the alternatively spliced isoforms that emerged from the transcript-specific analysis. **B**. Genes that populate the biological processes "*Spliceosome*" and "*Protein processing in ER*" and are alternatively spliced upon loss of Sox9. **C**. The distribution of alternative splicing events in the mouse reference genome. Total number of all possible events is shown. **D**. A subset of genes common to both rMATS and Sleuth analyses is shown. **E**. Immunostaining for Srsf5 in control and *Ins-Cre;Sox9-/-* islets at one year. Size bar, 50μm. **F**. PCR validation of the abundance of transcripts with increased levels of intron 5 inclusion in Srsf5 between control (n=4 animals) and knockout (n=4 animals) islets in the *MIP-Cre^{ERT};Sox9-/-* cohort. Primer locations for each product are shown. Quantification of the semi-quantitative PCR is shown. Error bars represent S.E.M. using a two-tailed Student's t test. P values are indicated. **G**. Differential gene expression of *Srsf6* abundance between control (white, n=4) and knockout (*Ins-Cre;Sox9-/-,* green, n=4) samples. **H**. Transcript level analyses of reads from the RNA-seq data depicting the abundance of the *Srsf6* protein-coding transcript (blue box plot) and non-coding isoforms (orange box plots) in control (n=4) and *Ins-Cre;Sox9-/-* (n=4) islets. P values are indicated. **I-L**. Estimated counts of protein coding (blue box plot) and non-protein coding (orange box plot) for *Hsph1*, *Syvn1*, *Hsp90b1*, and *Dnaja1* in control (n=4) and *Ins-Cre;Sox9-/-* (n=4) islets. For alternative splicing, p values were generated by sleuth or rMATs as per the software parameters. Data for **H-L** are presented as box plots (center line at the median, upper bound at 75th percentile, lower bound at 25th percentile) with whiskers at minimum and maximum values. P values are indicated.

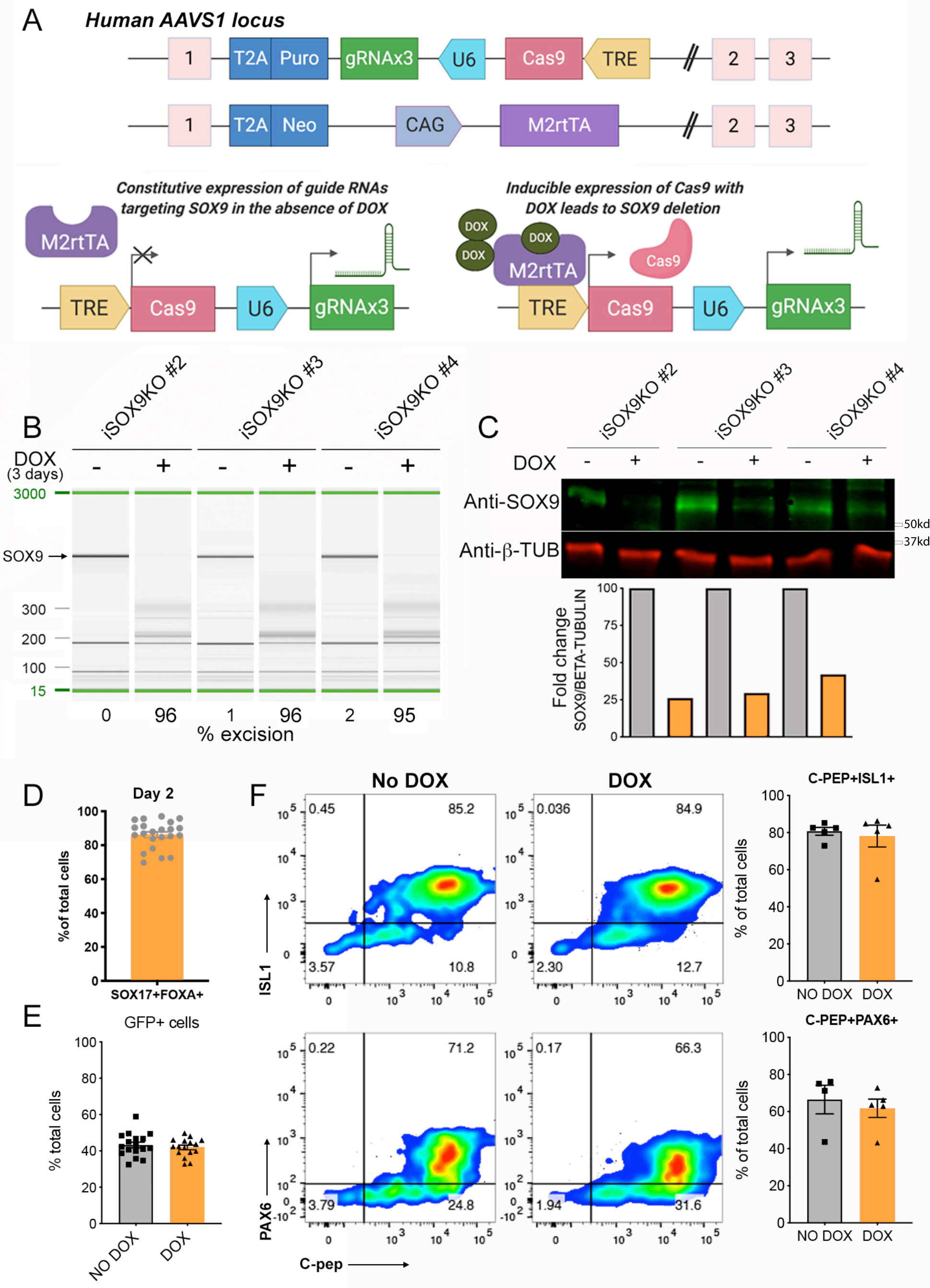

Figure S4. Loss of SOX9 in human beta cells. A. A schematic representation of the generation of human stem cell lines knocked out for SOX9 using CRISPR. Using an inducible system, the human stem cell line INS-GFP was transfected with plasmid constructs for insertion into the AAVS1 locus. Addition of doxycycline (DOX) led to the induction of Cas9 expression. Three guide RNAs that targeted SOX9 were inserted in tandem under the U6 promoter and were constitutively expressed from the constructs. **B**. Three independent clonal lines were treated with DOX for 3 days, and a T7 endonuclease assay carried out to determine percent excision of the SOX9 locus. The data show three independent experiments. **C**. Western blotting on extracts from iSOX9KO cells treated with DOX from day 13 to day 20 of differentiation. No DOX controls are shown in the grey bar graph and DOX-treated cells are shown in orange. >75% knockdown was achieved. **D**. Differentiation of iSOX9KO line #2 to day 2. N=22 independent differentiations. Data are presented as mean values +/- SEM. **E**. Quantification of GFP-positive cells at day 20 of differentiation with or without DOX. N=17 differentiations for No DOX, N=16 differentiations with DOX. Data are presented as mean values +/- SEM. **F**. Flow analysis of beta cell markers in C-PEP positive cells with or without DOX. C-PEP/ISL1 staining, N=5 independent differentiations for both groups; C-PEP/PAX6, N=4 independent differentiations for No DOX, N=5 independent differentiations for with DOX. Data are presented as mean values +/- SEM.

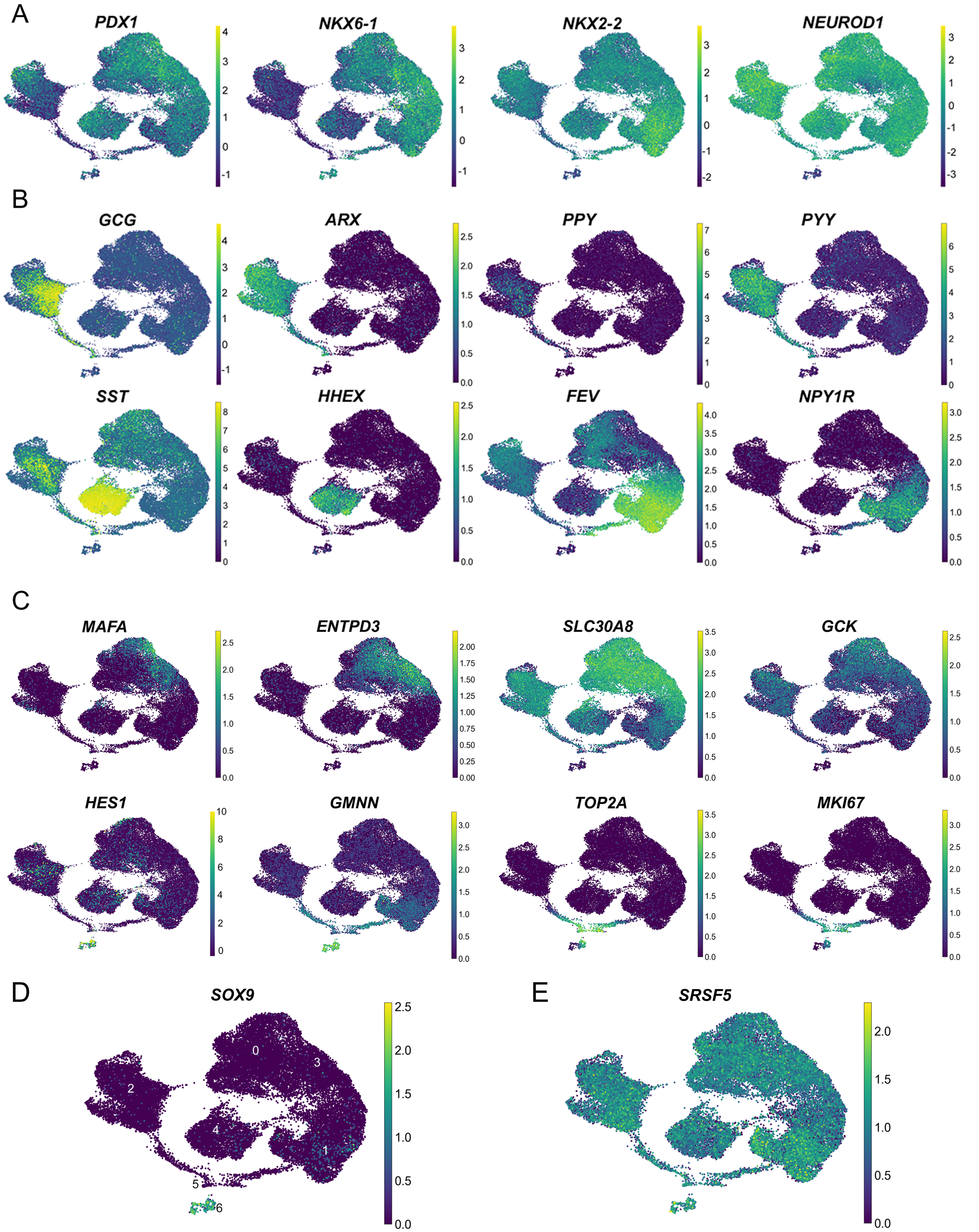

Figure S5. UMAP plots for genes from single cell sequencing of human cells. A. UMAP plots of canonical beta cell markers generated from the single cell sequencing data. **B**. UMAP plots of non-beta cell genes are shown. **C.** UMAP plots of markers that define specific populations. *SOX9* (**D**) and *SRSF5* (**E**) UMAP plots are also shown. **F**. A representation of the proportions of populations within the different clusters shown in figure 5D.

Figure S6. KEGG pathways showing the localization of genes dysregulated upon Sox9 loss in the rodent model.

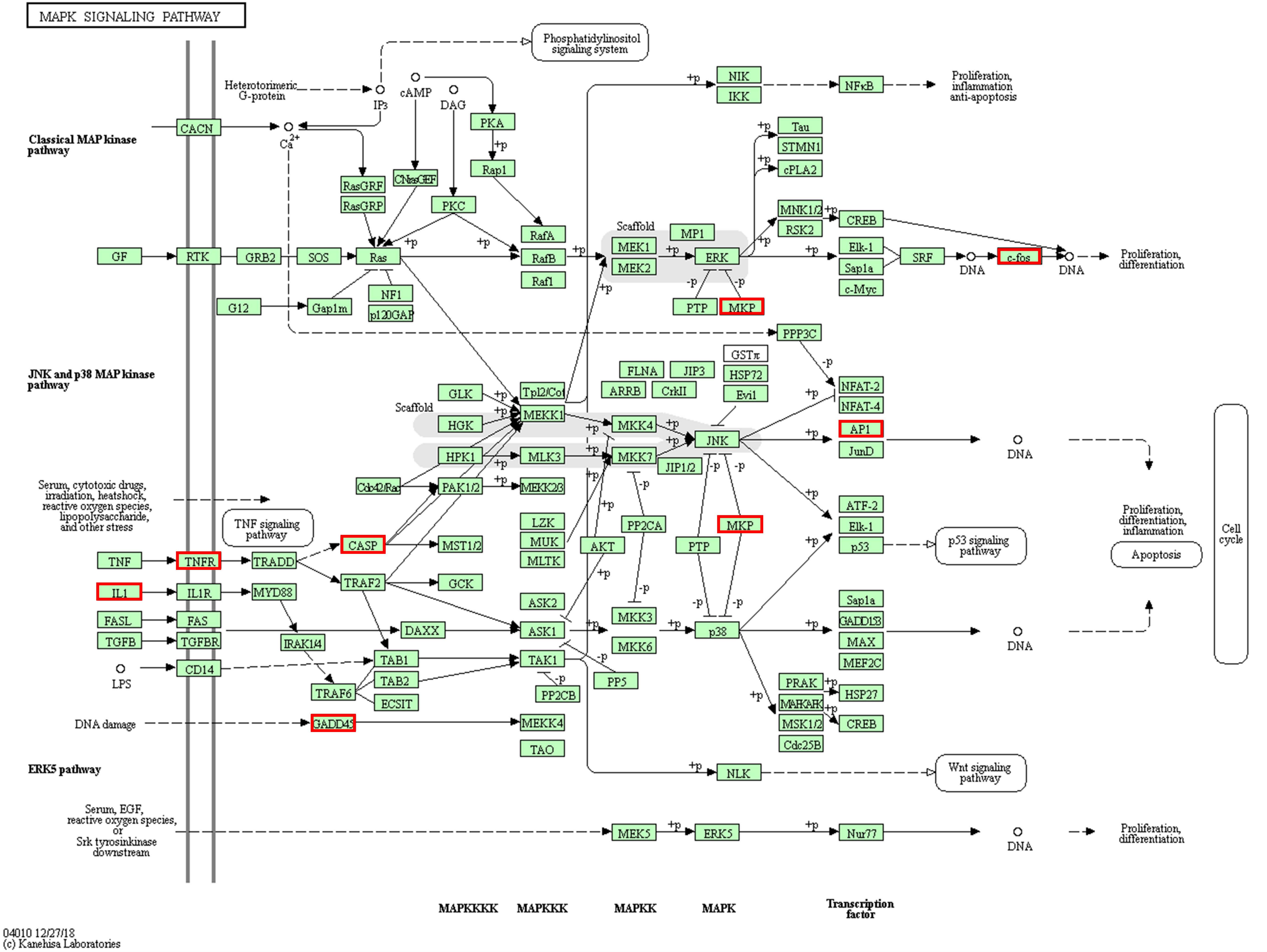

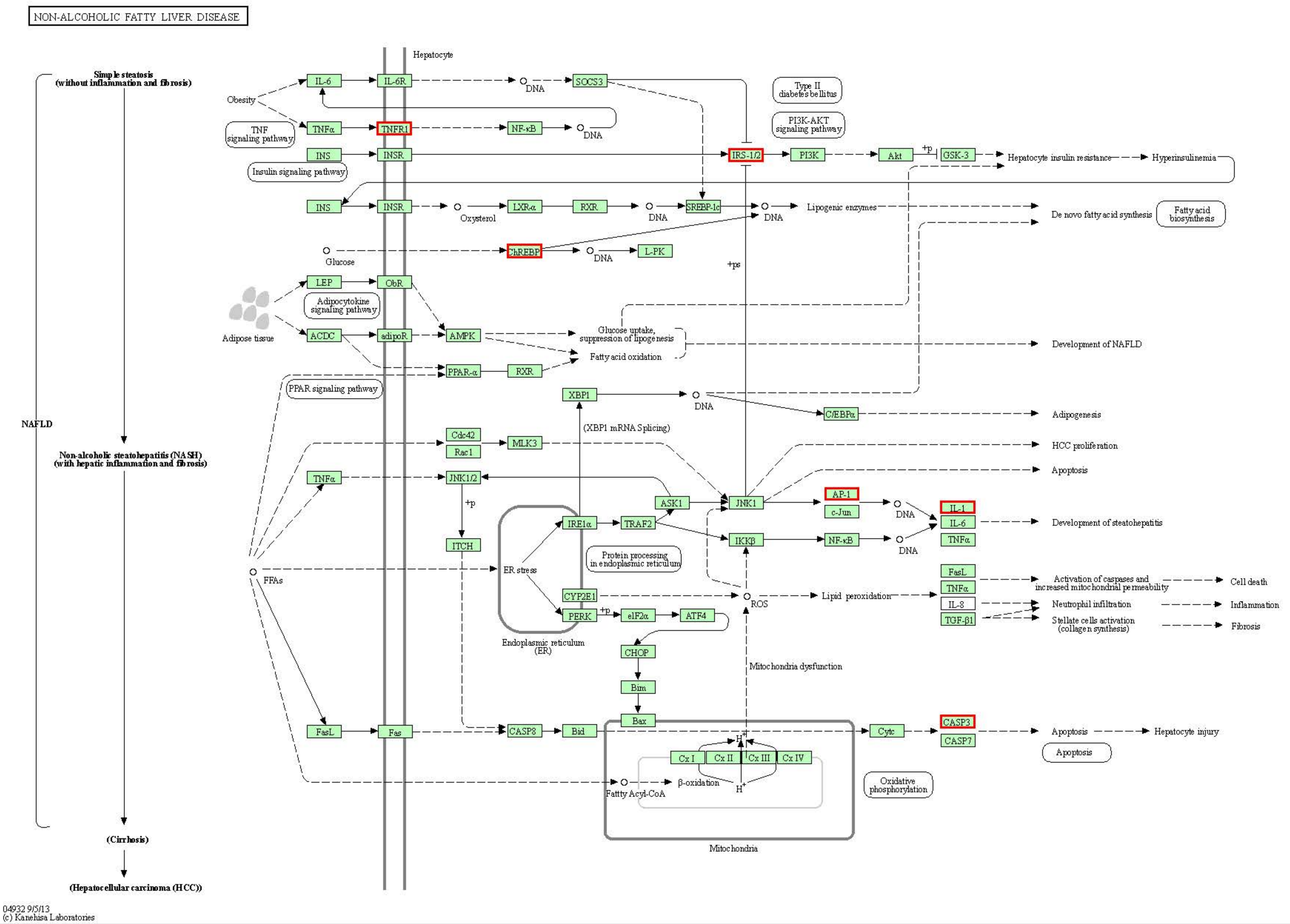

NON-ALCOHOLIC FATTY LIVER DISEASE
Hepatocyte
Simple steatosis (without inflammation and fibrosis)
NAFLD
Non-alcoholic steatohepatitis (NASH) (with hepatic inflammation and fibrosis)
(Cirrhosis)
(Hepatocellular carcinoma (HCC))
Obesity
IL-6
IL-6R
DNA
SOCS3
TNF signaling pathway
TNFα
TNFR1
NF-κB
INS
INSR
Insulin signaling pathway
IRS-1/2
PI3K
Akt
+p
GSK-3
Hepatocyte insulin resistance
Hyperinsulinemia
Type II diabetes mellitus
PI3K-AKT signaling pathway
Oxysterol
LXR-α
RXR
SREBP-1c
Lipogenic enzymes
De novo fatty acid synthesis
Fatty acid biosynthesis
Glucose
ChREBP
L-PK
+ps
Adipose tissue
LEP
ObR
Adipocytokine signaling pathway
ACDC
adipoR
AMPK
Glucose uptake, suppression of lipogenesis
Fatty acid oxidation
Development of NAFLD
PPAR-α
PPAR signaling pathway
XBP1
(XBP1 mRNA Splicing)
C/EBPα
Adipogenesis
Cdc42
Rac1
MLK3
HCC proliferation
Apoptosis
JNK1/2
ASK1
JNK1
AP-1
c-Jun
IL-1
Development of steatohepatitis
ITCH
IRE1α
TRAF2
IKKβ
Protein processing in endoplasmic reticulum
FFAs
ER stress
CYP2E1
ROS
Lipid peroxidation
FasL
Activation of caspases and increased mitochondrial permeability
Cell death
IL-8
Neutrophil infiltration
Inflammation
TGF-β1
Stellate cells activation (collagen synthesis)
Fibrosis
PERK
eIF2α
ATF4
Endoplasmic reticulum (ER)
CHOP
Mitochondria dysfunction
Bim
Bax
Fas
CASP8
Bid
Cytc
CASP3
CASP7
Hepatocyte injury
Cx I
Cx II
Cx III
Cx IV
Fatty Acyl-CoA
β-oxidation
Oxidative phosphorylation
Mitochondria
04932 9/5/13
(c) Kanehisa Laboratories

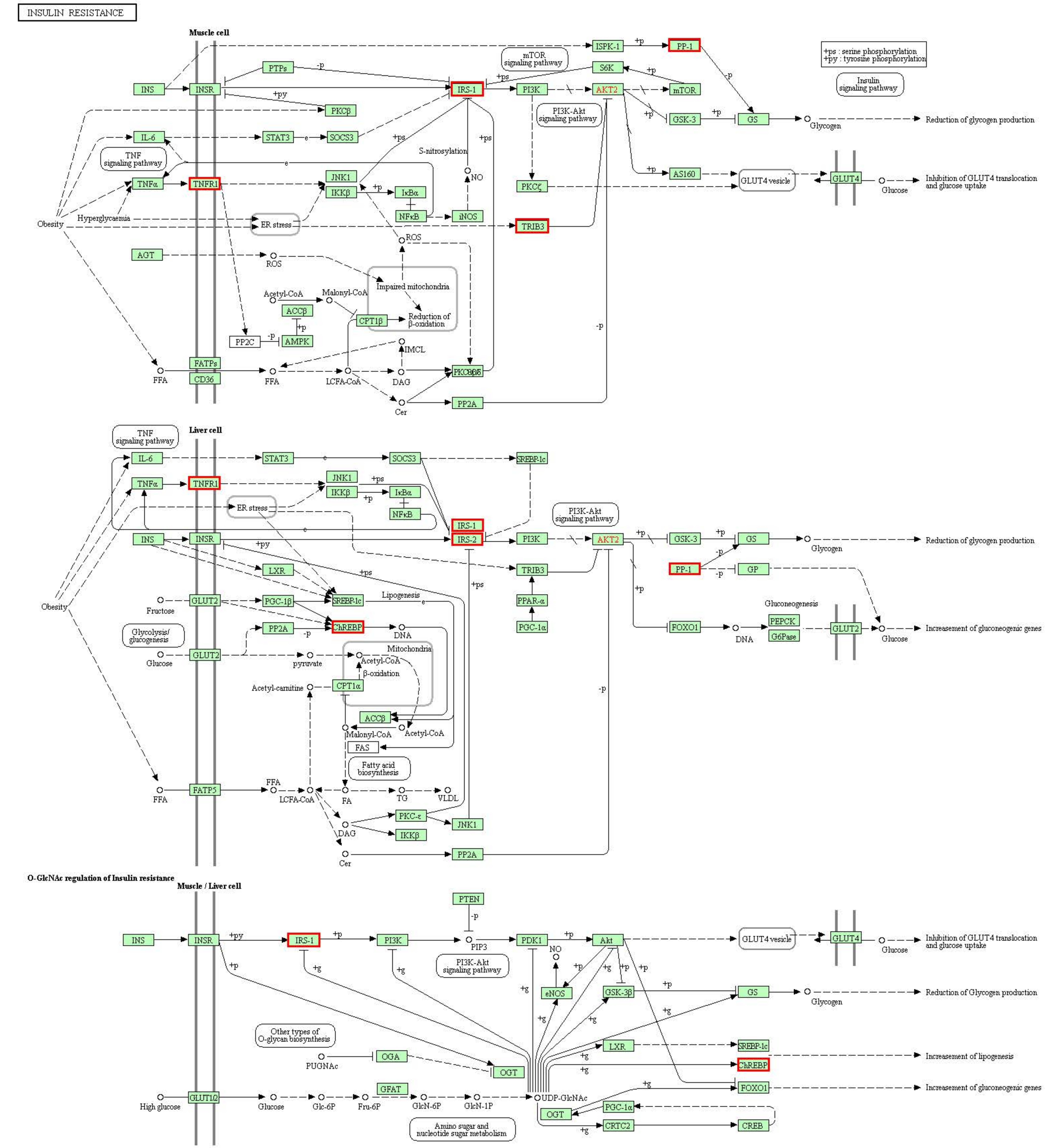
INSULIN RESISTANCE
Muscle cell
INS
INSR
PTPs
PKCβ
IL-6
STAT3
SOCS3
TNF signaling pathway
TNFα
TNFR1
JNK1
IKKβ
IκBα
NFκB
iNOS
NO
S-nitrosylation
IRS-1
PI3K
AKT2
mTOR
S6K
ISPK-1
PP-1
mTOR signaling pathway
PI3K-Akt signaling pathway
GSK-3
GS
Glycogen
Reduction of glycogen production
AS160
GLUT4 vesicle
GLUT4
Glucose
Inhibition of GLUT4 translocation and glucose uptake
PKCζ
TRIB3
+ps : serine phosphorylation
+py : tyrosine phosphorylation
Insulin signaling pathway
Obesity
Hyperglycaemia
ER stress
AGT
ROS
Impaired mitochondria
Acetyl-CoA
Malonyl-CoA
ACCβ
CPT1β
Reduction of β-oxidation
PP2C
AMPK
IMCL
FATPs
CD36
FFA
LCFA-CoA
DAG
PKCθ/δ
Cer
PP2A
Liver cell
SREBP-1c
IRS-2
GP
LXR
GLUT2
PGC-1β
Lipogenesis
ChREBP
DNA
Fructose
Glycolysis/ glucogenesis
pyruvate
Mitochondria
β-oxidation
CPT1α
Acetyl-carnitine
FAS
Fatty acid biosynthesis
FATP5
FA
TG
VLDL
PKC-ε
PPAR-α
PGC-1α
FOXO1
Gluconeogenesis
PEPCK
G6Pase
Increasement of gluconeogenic genes
O-GlcNAc regulation of Insulin resistance
Muscle / Liver cell
PTEN
PIP3
PDK1
Akt
eNOS
GSK-3β
Reduction of Glycogen production
Other types of O-glycan biosynthesis
PUGNAc
OGA
OGT
Increasement of lipogenesis
High glucose
GLUT1/2
Glc-6P
Fru-6P
GFAT
GlcN-6P
GlcN-1P
UDP-GlcNAc
CRTC2
CREB
Amino sugar and nucleotide sugar metabolism
+p
-p
+py
+ps
+g
e

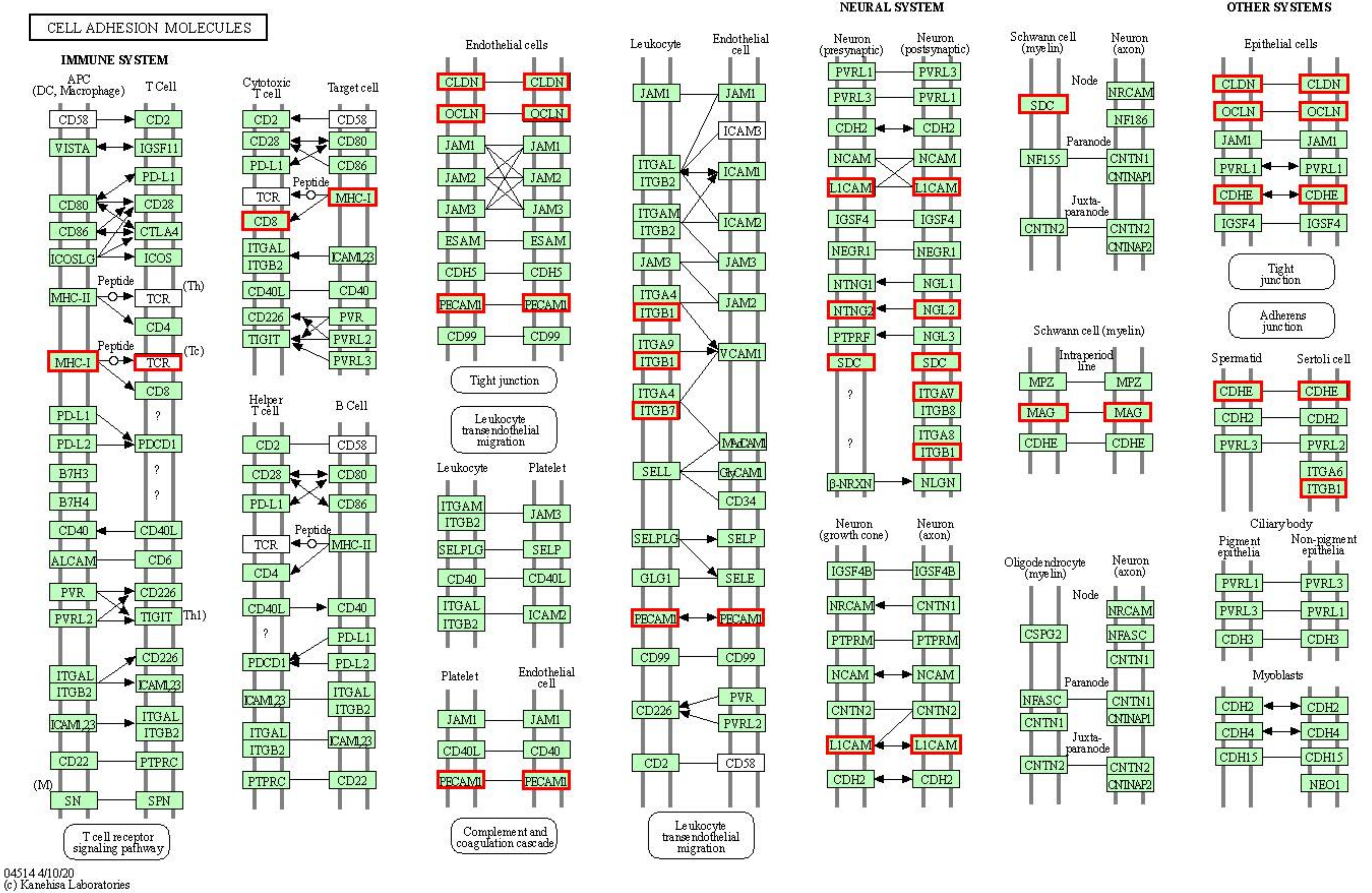
CELL ADHESION MOLECULES
IMMUNE SYSTEM
NEURAL SYSTEM
OTHER SYSTEMS
APC (DC, Macrophage)
T Cell
Cytotoxic T cell
Target cell
Helper T cell
B Cell
Endothelial cells
Leukocyte
Platelet
Endothelial cell
Neuron (presynaptic)
Neuron (postsynaptic)
Neuron (growth cone)
Neuron (axon)
Schwann cell (myelin)
Oligodendrocyte (myelin)
Epithelial cells
Spermatid
Sertoli cell
Ciliary body
Pigment epithelia
Non-pigment epithelia
Myoblasts
Node
Paranode
Juxta-paranode
Intraperiod line
Peptide
(Th)
(Tc)
Th1)
(M)
CD58 CD2 VISTA IGSF11 PD-L1 CD80 CD28 CD86 CTLA4 ICOSLG ICOS MHC-II TCR CD4 MHC-I CD8 PD-L2 PDCD1 B7H3 B7H4 CD40 CD40L ALCAM CD6 PVR CD226 PVRL2 TIGIT ITGAL ITGB2 ICAM1,2,3 CD22 PTPRC SN SPN
PVRL3 CD40L CD40 PD-L1 PD-L2
CLDN OCLN JAM1 JAM2 JAM3 ESAM CDH5 PECAM1 CD99
ITGAM ITGB2 JAM3 SELPLG SELP ITGAL ICAM2
ICAM3 ICAM1 ICAM2 ITGA4 ITGB1 ITGA9 VCAM1 ITGB7 MAdCAM1 SELL GlyCAM1 CD34 SELE GLG1 PVRL2 CD58
PVRL1 PVRL3 CDH2 NCAM L1CAM IGSF4 NEGR1 NTNG1 NGL1 NTNG2 NGL2 PTPRF NGL3 SDC ITGAV ITGB8 ITGA8 ITGB1 β-NRXN NLGN
IGSF4B NRCAM CNTN1 PTPRM CNTN2
NF186 NF155 CNTNAP1 CNTNAP2 MPZ MAG CDHE CSPG2 NFASC
ITGA6 CDH3 CDH4 CDH15 NEO1
Tight junction
Adherens junction
T cell receptor signaling pathway
Leukocyte transendothelial migration
Complement and coagulation cascade
04514 4/10/20
(c) Kanehisa Laboratories

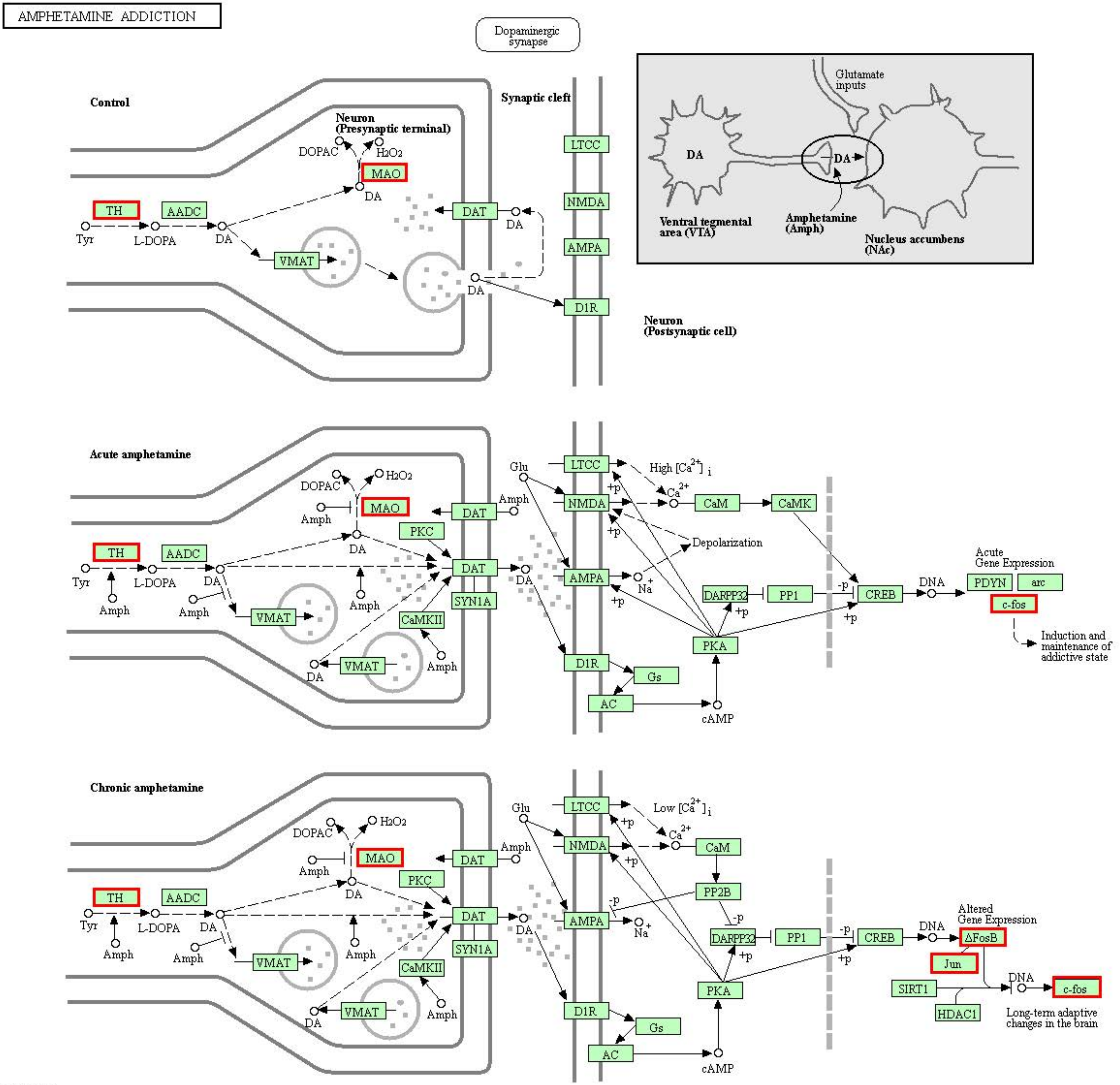

05031 10/28/19
(c) Kanehisa Laboratories

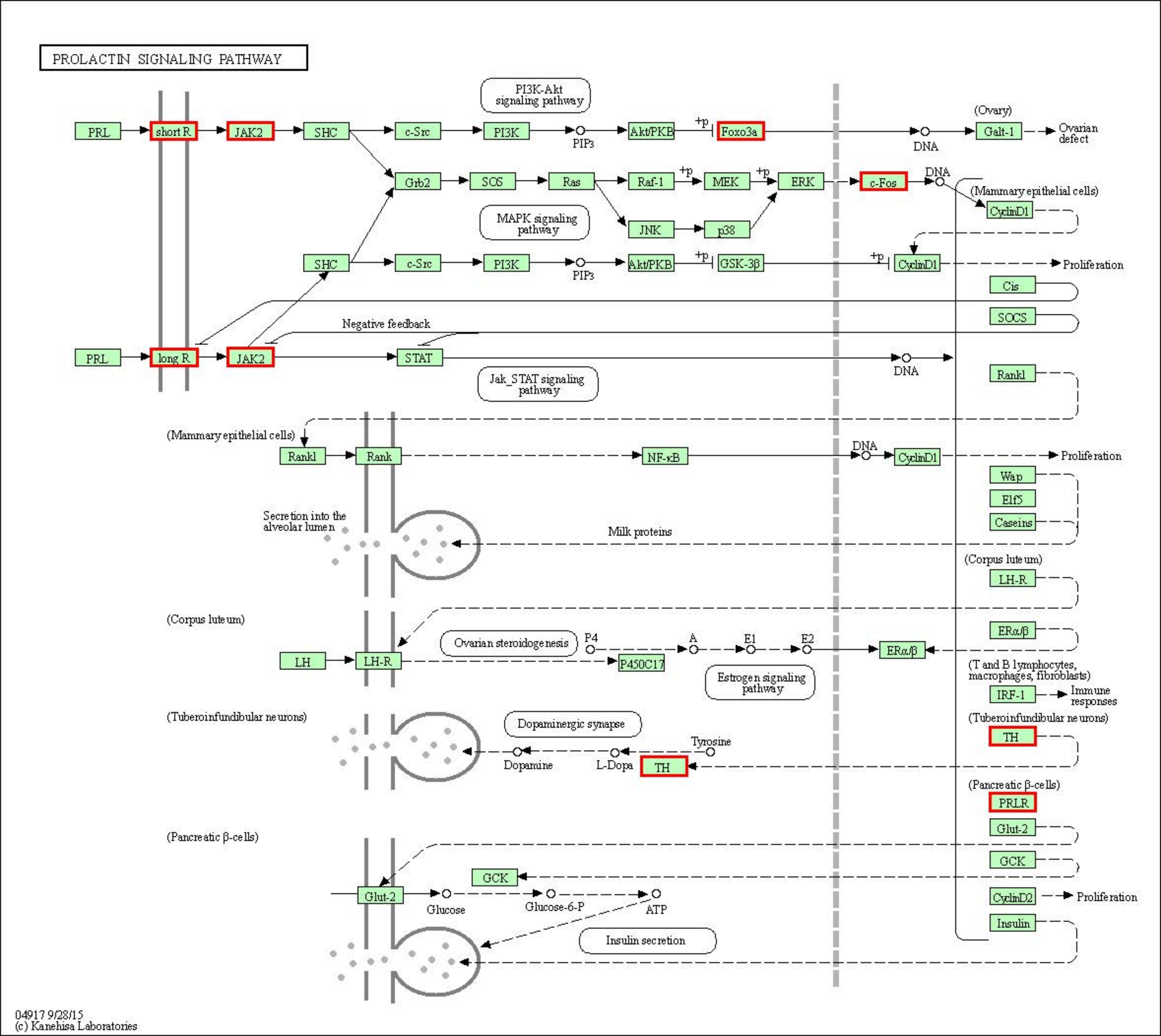
PROLACTIN SIGNALING PATHWAY
PI3K-Akt signaling pathway
PRL
short R
JAK2
SHC
c-Src
PI3K
PIP3
Akt/PKB
+p
Foxo3a
DNA
(Ovary)
Galt-1
Ovarian defect
Grb2
SOS
Ras
Raf-1
MEK
ERK
c-Fos
(Mammary epithelial cells)
CyclinD1
MAPK signaling pathway
JNK
p38
GSK-3β
Proliferation
Cis
SOCS
Negative feedback
long R
STAT
Jak_STAT signaling pathway
Rankl
Rank
NF-κB
Wap
Elf5
Caseins
Secretion into the alveolar lumen
Milk proteins
(Corpus luteum)
LH-R
ERα/β
LH
Ovarian steroidogenesis
P4
A
E1
E2
P450C17
Estrogen signaling pathway
(T and B lymphocytes, macrophages, fibroblasts)
IRF-1
Immune responses
(Tuberoinfundibular neurons)
TH
Dopaminergic synapse
Tyrosine
Dopamine
L-Dopa
(Pancreatic β-cells)
PRLR
Glut-2
GCK
Glucose
Glucose-6-P
ATP
CyclinD2
Insulin
Insulin secretion
04917 9/28/15
(c) Kanehisa Laboratories

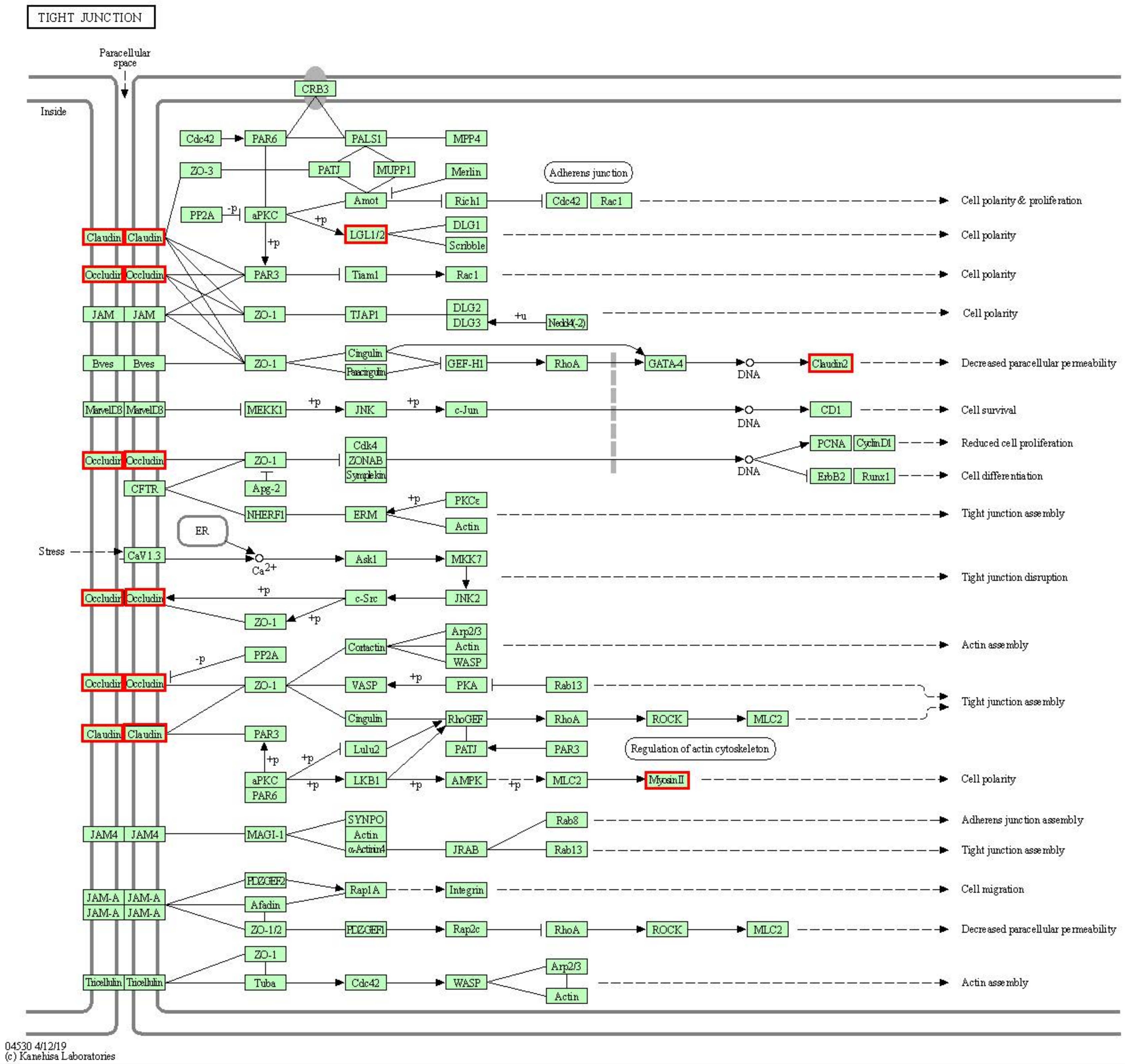
TIGHT JUNCTION
Paracellular space
Inside
CRB3
Cdc42
PAR6
PALS1
MPP4
ZO-3
PATJ
MUPP1
Merlin
Adherens junction
PP2A
-p
aPKC
Amot
Rich1
Cdc42
Rac1
Cell polarity & proliferation
+p
LGL1/2
DLG1
Scribble
Cell polarity
Claudin
Claudin
+p
Occludin
Occludin
PAR3
Tiam1
Rac1
Cell polarity
JAM
JAM
ZO-1
TJAP1
DLG2
DLG3
+u
Nedd4(-2)
Cell polarity
Bves
Bves
ZO-1
Cingulin
Paracingulin
GEF-H1
RhoA
GATA-4
DNA
Claudin2
Decreased paracellular permeability
MarvelD3
MarvelD3
MEKK1
+p
JNK
+p
c-Jun
DNA
CD1
Cell survival
Occludin
Occludin
ZO-1
Cdk4
ZONAB
Symplekin
DNA
PCNA
CyclinD1
Reduced cell proliferation
Apg-2
ErbB2
Runx1
Cell differentiation
CFTR
NHERF1
ERM
+p
PKCε
Actin
Tight junction assembly
ER
Stress
CaV1.3
Ca2+
Ask1
MKK7
Tight junction disruption
Occludin
Occludin
+p
c-Src
JNK2
ZO-1
+p
Cortactin
Arp2/3
Actin
WASP
Actin assembly
-p
PP2A
Occludin
Occludin
ZO-1
VASP
+p
PKA
Rab13
Tight junction assembly
Cingulin
RhoGEF
RhoA
ROCK
MLC2
Claudin
Claudin
PAR3
Lulu2
PATJ
PAR3
Regulation of actin cytoskeleton
+p
+p
aPKC
PAR6
+p
LKB1
+p
AMPK
+p
MLC2
Myosin II
Cell polarity
SYNPO
Actin
α-Actinin4
Rab8
Adherens junction assembly
JAM4
JAM4
MAGI-1
JRAB
Rab13
Tight junction assembly
PDZGEF2
Rap1A
Integrin
Cell migration
JAM-A
JAM-A
JAM-A
JAM-A
Afadin
ZO-1/2
PDZGEF1
Rap2c
RhoA
ROCK
MLC2
Decreased paracellular permeability
ZO-1
Tricellulin
Tricellulin
Tuba
Cdc42
WASP
Arp2/3
Actin
Actin assembly
04530 4/12/19
(c) Kanehisa Laboratories

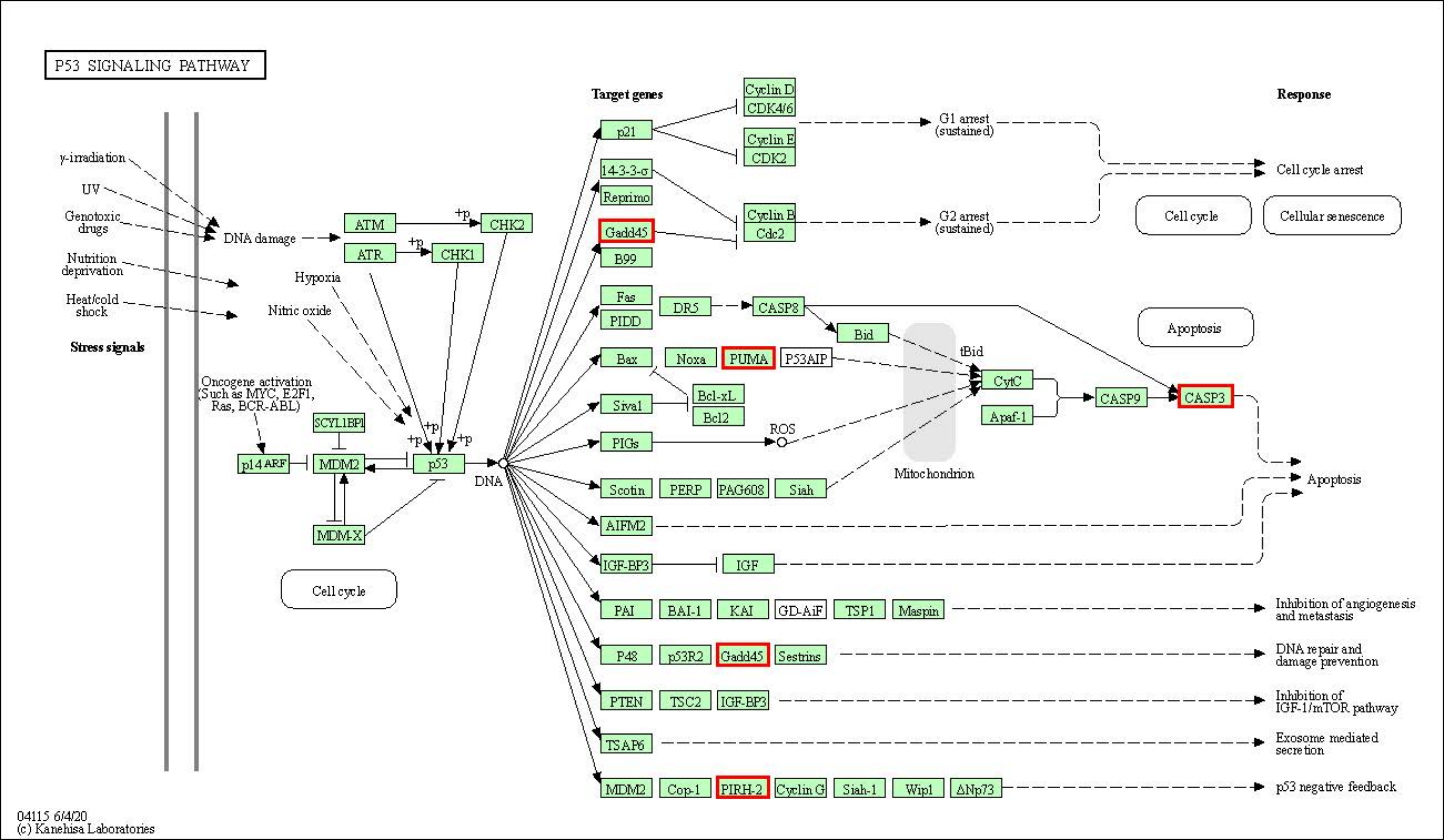

P53 SIGNALING PATHWAY
Target genes
Response
Stress signals
γ-irradiation
UV
Genotoxic drugs
Nutrition deprivation
Heat/cold shock
DNA damage
Hypoxia
Nitric oxide
ATM
ATR
CHK2
CHK1
+p
Oncogene activation (Such as MYC, E2F1, Ras, BCR-ABL)
p14ARF
MDM2
SCYL1BP1
MDM-X
p53
DNA
Cell cycle
p21
14-3-3-σ
Reprimo
Gadd45
B99
Cyclin D
CDK4/6
Cyclin E
CDK2
Cyclin B
Cdc2
G1 arrest (sustained)
G2 arrest (sustained)
Cell cycle arrest
Cell cycle
Cellular senescence
Fas
PIDD
DR5
CASP8
Bid
tBid
Bax
Noxa
PUMA
P53AIP
Bcl-xL
Bcl2
Sival
PIGs
ROS
CytC
Apaf-1
CASP9
CASP3
Mitochondrion
Apoptosis
Scotin
PERP
PAG608
Siah
AIFM2
IGF-BP3
IGF
PAI
BAI-1
KAI
GD-AiF
TSP1
Maspin
Inhibition of angiogenesis and metastasis
P48
p53R2
Gadd45
Sestrins
DNA repair and damage prevention
PTEN
TSC2
IGF-BP3
Inhibition of IGF-1/mTOR pathway
TSAP6
Exosome mediated secretion
MDM2
Cop-1
PIRH-2
Cyclin G
Siah-1
Wip1
ΔNp73
p53 negative feedback
04115 6/4/20
(c) Kanehisa Laboratories

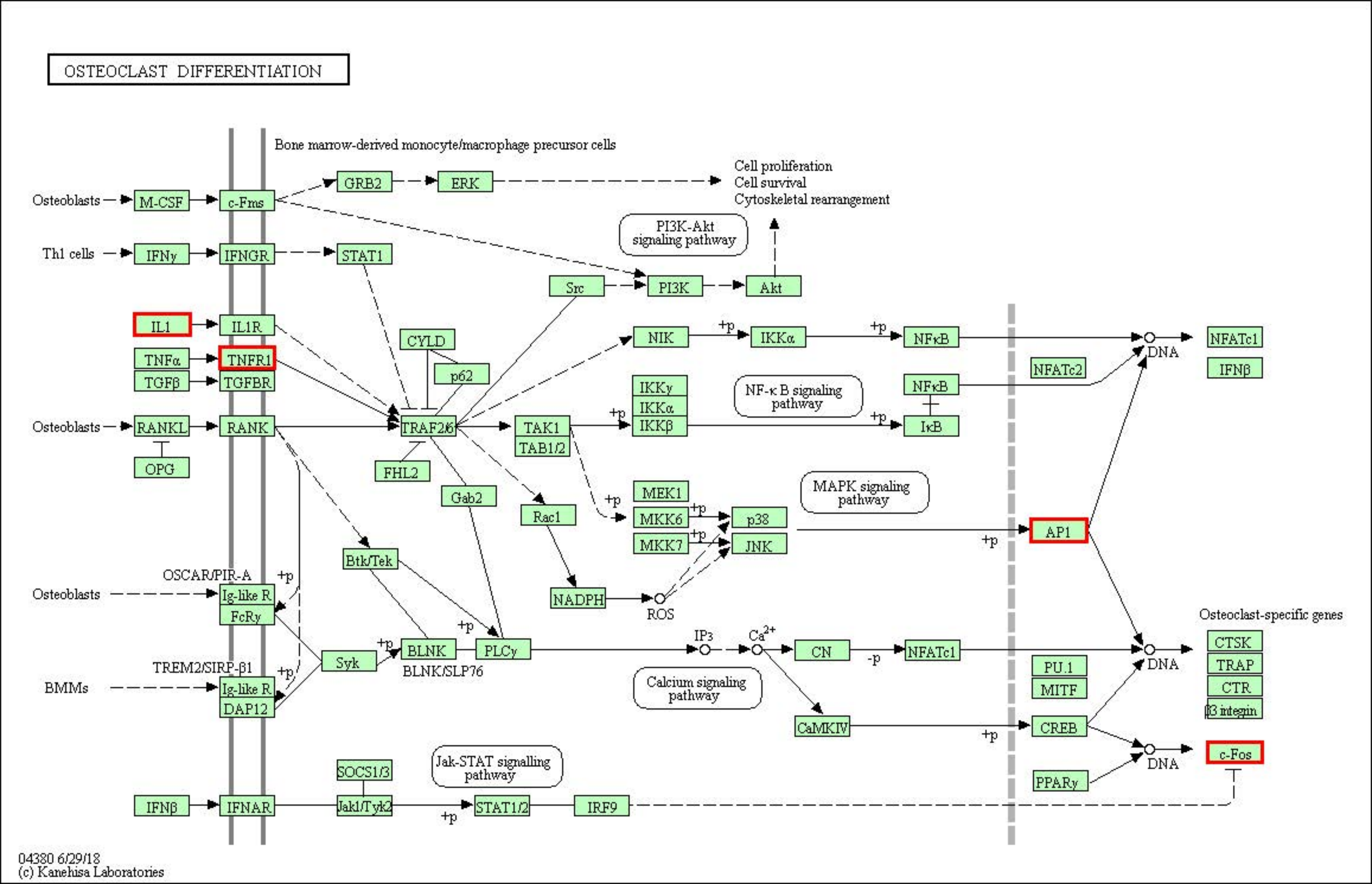

OSTEOCLAST DIFFERENTIATION
Bone marrow-derived monocyte/macrophage precursor cells
Osteoblasts
M-CSF
c-Fms
GRB2
ERK
Cell proliferation
Cell survival
Cytoskeletal rearrangement
PI3K-Akt signaling pathway
Th1 cells
IFNγ
IFNGR
STAT1
Src
PI3K
Akt
IL1
IL1R
TNFα
TNFR1
TGFβ
TGFBR
CYLD
p62
NIK
IKKα
NFκB
NFATc1
NFATc2
IFNβ
DNA
IKKγ
IKKα
IKKβ
NF-κ B signaling pathway
NFκB
IκB
Osteoblasts
RANKL
RANK
TRAF2/6
TAK1
TAB1/2
OPG
FHL2
Gab2
MEK1
MKK6
MKK7
p38
JNK
MAPK signaling pathway
Rac1
AP1
+p
OSCAR/PIR-A
Osteoblasts
Ig-like R
FcRγ
Btk/Tek
NADPH
ROS
Osteoclast-specific genes
Syk
BLNK
PLCγ
BLNK/SLP76
IP3
Ca2+
CN
NFATc1
-p
DNA
CTSK
TRAP
CTR
β3 integrin
PU.1
MITF
TREM2/SIRP-β1
BMMs
Ig-like R
DAP12
Calcium signaling pathway
CaMKIV
CREB
DNA
c-Fos
PPARγ
SOCS1/3
Jak-STAT signalling pathway
IFNβ
IFNAR
Jak1/Tyk2
STAT1/2
IRF9
04380 6/29/18
(c) Kanehisa Laboratories

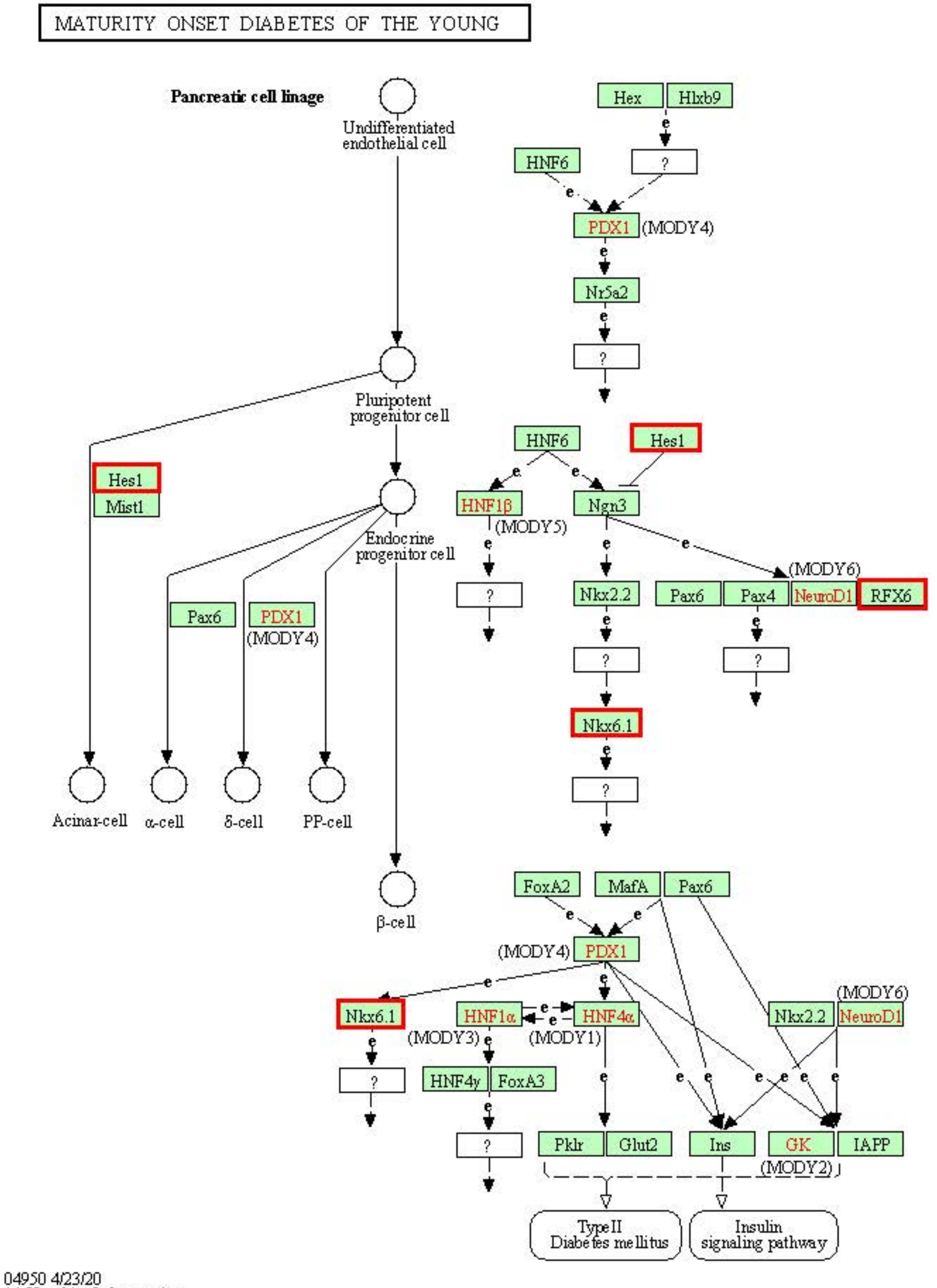

04950 4/23/20
(c) Kanehisa Laboratories

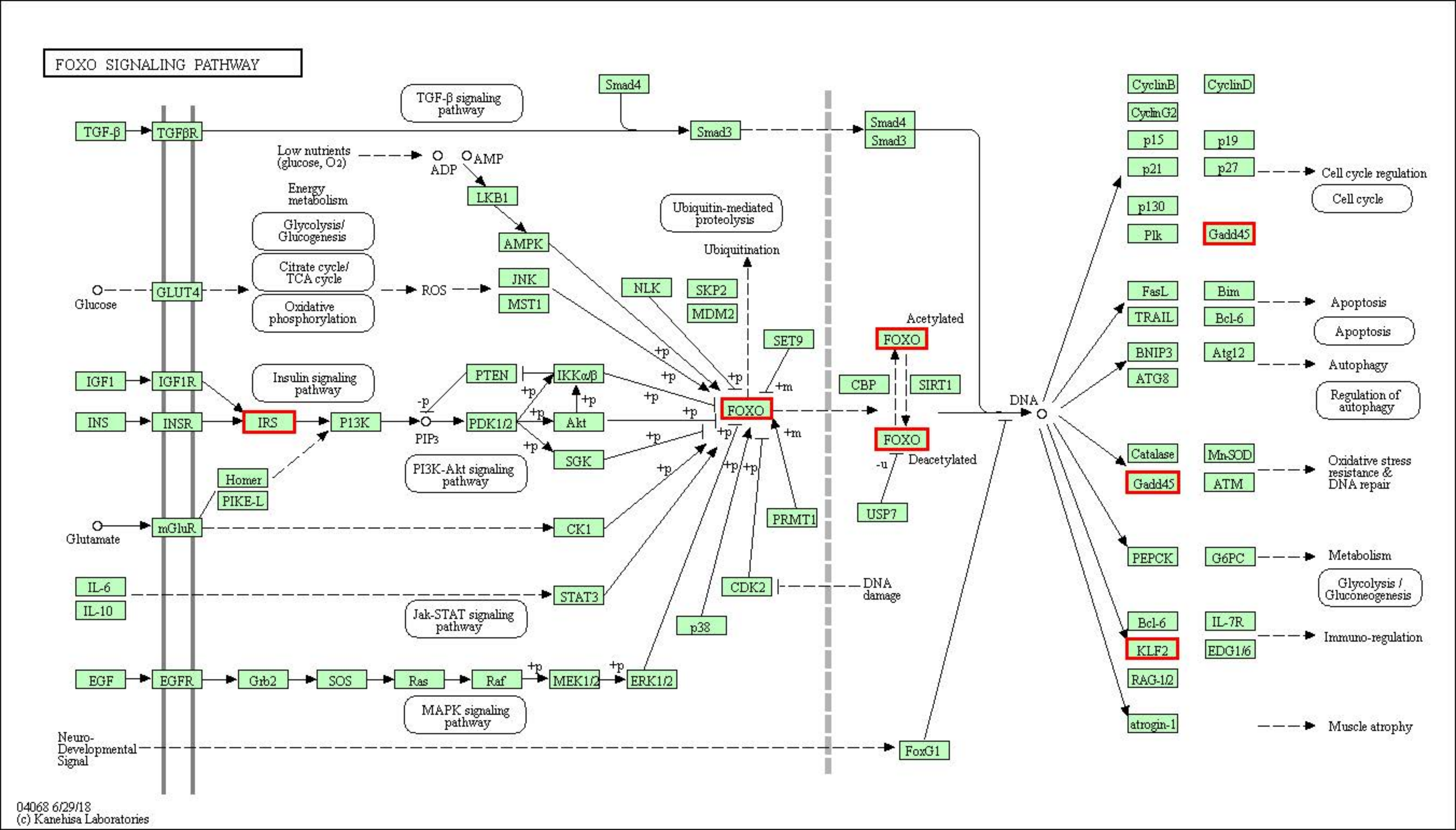
FOXO SIGNALING PATHWAY
TGF-β signaling pathway
Smad4
TGF-β
TGFβR
Smad3
Smad4
Smad3
Low nutrients (glucose, O2)
ADP
AMP
LKB1
Energy metabolism
Glycolysis/ Glucogenesis
Citrate cycle/ TCA cycle
Oxidative phosphorylation
AMPK
Ubiquitin-mediated proteolysis
Ubiquitination
Glucose
GLUT4
ROS
JNK
MST1
NLK
SKP2
MDM2
SET9
Acetylated
FOXO
CBP
SIRT1
IGF1
IGF1R
Insulin signaling pathway
PTEN
IKKα/β
INS
INSR
IRS
PI3K
PIP3
PDK1/2
Akt
SGK
FOXO
FOXO
Deacetylated
USP7
PRMT1
PI3K-Akt signaling pathway
Homer
PIKE-L
Glutamate
mGluR
CK1
IL-6
IL-10
STAT3
Jak-STAT signaling pathway
CDK2
DNA damage
p38
EGF
EGFR
Grb2
SOS
Ras
Raf
MEK1/2
ERK1/2
MAPK signaling pathway
Neuro-Developmental Signal
FoxG1
DNA
CyclinB
CyclinD
CyclinG2
p15
p19
p21
p27
Cell cycle regulation
Cell cycle
p130
Plk
Gadd45
FasL
Bim
TRAIL
Bcl-6
Apoptosis
Apoptosis
BNIP3
Atg12
ATG8
Autophagy
Regulation of autophagy
Catalase
Mn-SOD
Gadd45
ATM
Oxidative stress resistance & DNA repair
PEPCK
G6PC
Metabolism
Glycolysis / Gluconeogenesis
Bcl-6
IL-7R
KLF2
EDG1/6
Immuno-regulation
RAG-1/2
atrogin-1
Muscle atrophy
04068 6/29/18
(c) Kanehisa Laboratories

Organism	Gene	Primer	Sequence
Mouse	Sox9	Forward	GCAGACCAGTACCCGCATCT
		Reverse	TTCAGCAGCCTCCAGAGCTT
	Ins1	Forward	CTGCTGGCCCTGCTTGC
		Reverse	GGGTCGAGGTGGGCCTT
	Ins2	Forward	CCTGCTGGCCCTGCTCTT
		Reverse	GGCTGGGTAGTGGTGGGTCTA
	Pdx1	Forward	AAATCCACCAAAGCTCACGC
		Reverse	GGGTTCCGCTGTGTAAGCA
	Nkx6.1	Forward	TCAGGTCAAGGTCTGGTTCCA
		Reverse	CGGTCTCCGAGTCCTGCTT
	Mafa	Forward	GCTGGTATCCATGTCCGTGC
		Reverse	TGTTTCAGTCGGATGACCTCC
	Neurod1	Forward	TCCGGTGCCGCTGC
		Reverse	GCGAATGGCTATCGAAAGACA
	Ucn3	Forward	AAGCCTCTCCCACAAGTTCTA
		Reverse	GAGGTGCGTTTGGTTGTCATC
	Glut2	Forward	AGTGGGCGGAATGGTCG
		Reverse	TGCTTTGATCCTTCCAAGTTTGT
	Cyclophilin A	Forward	TCACAGAATTATTCCAGGATTCATG
		Reverse	TGCCGCCAGTGCCATT

Primers used to detect gene expression in mouse islets

Gene	Primer	Sequence
Srsf5/SRSF5	Exon 5 Forward	5' AATGCTCCACCTGTAAGAACAGAA 3'
Srsf5/SRSF5	Intron 5 Reverse	5' CAACCAAGAGTGACCAAGACAAC 3'
Srsf5	Exon 6 Reverse	5' CCTTCATTTAGTTTAGGTCGATGTG 3'
SRSF5	Exon 8 Reverse	5′ CCCAGGGCAAGTTATTTACAGT 3′
GAPDH	Forward	5' GAAGGTGAAGGTCGGAGTC 3'
GAPDH	Reverse	5' GAAGATGGTGATGGGATTTC 3'

Primers used to detect the alternatively spliced products for Srsf5/SRSF5

FACS sequential gating/sorting strategies for Figure 5E

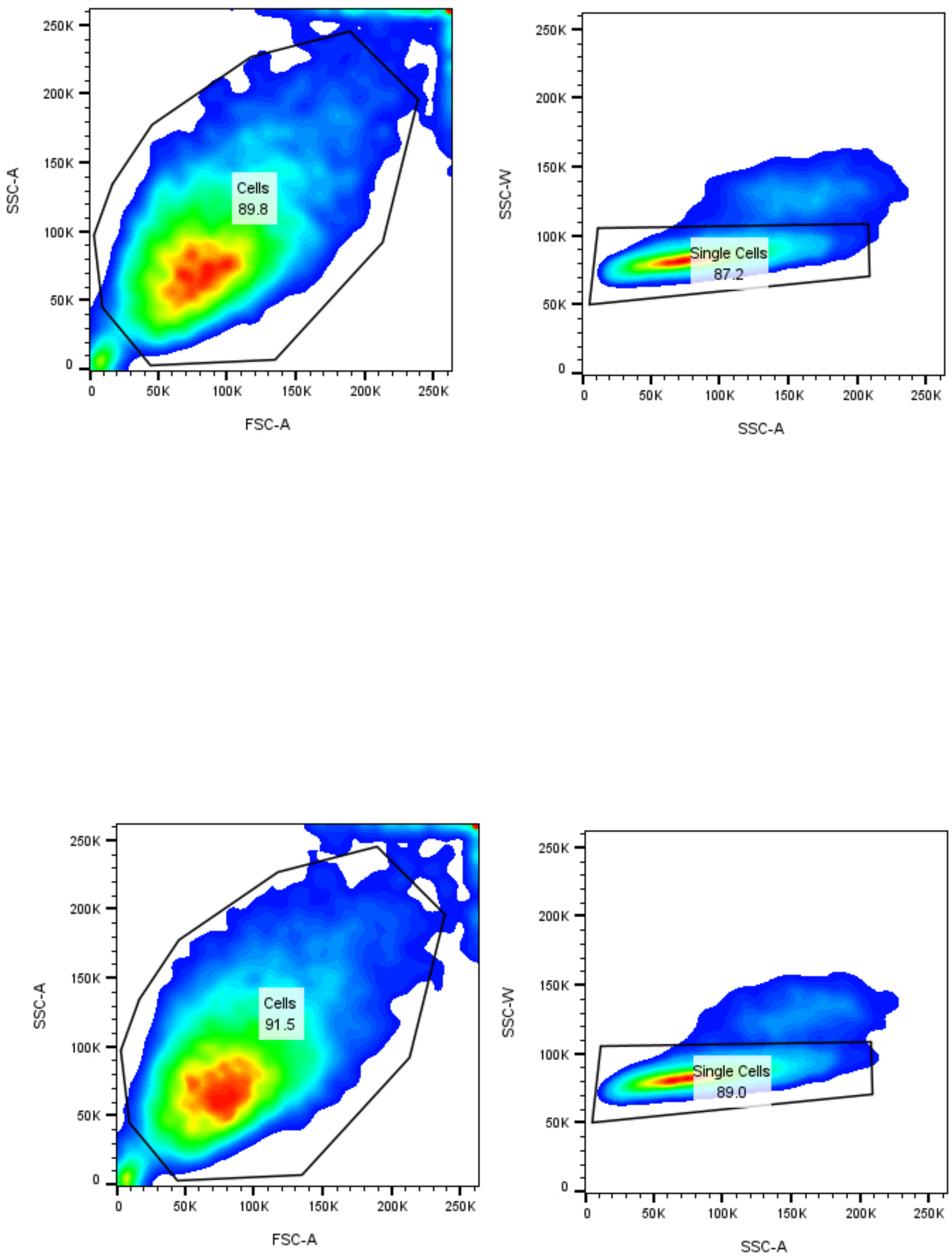

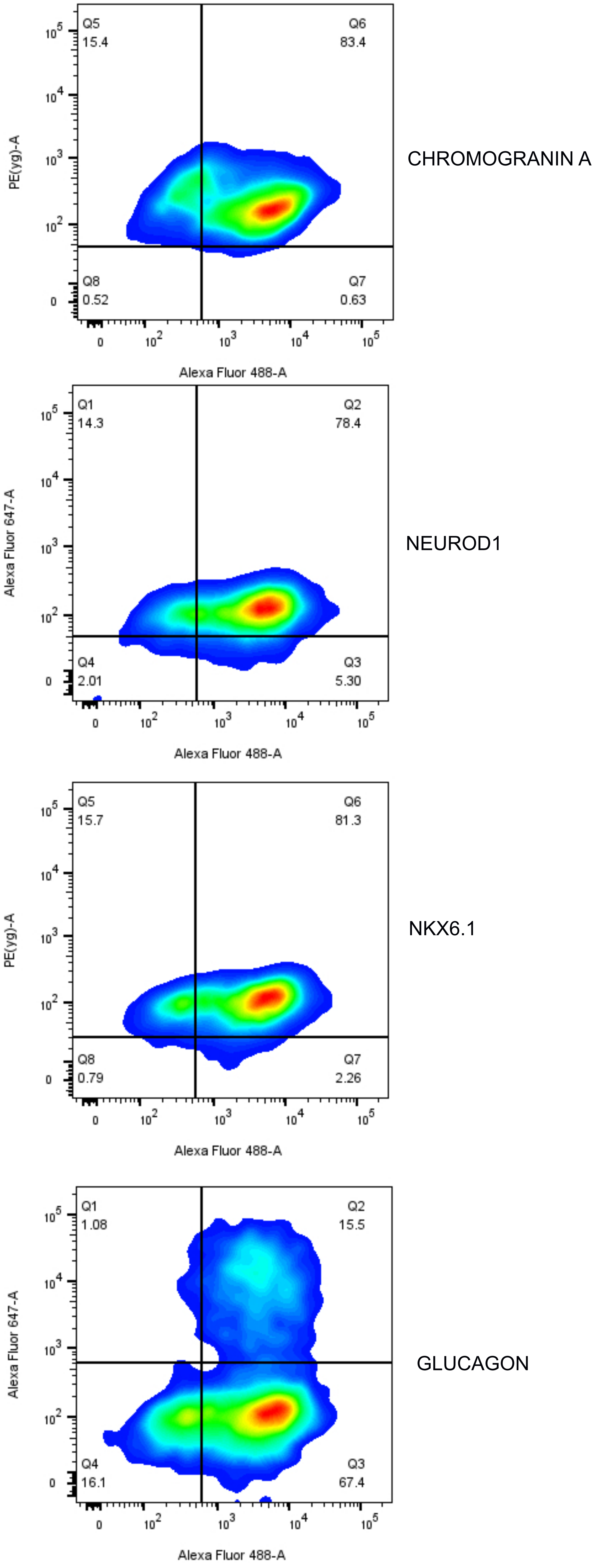

Supplement: Supplementary file 1 — Supplementary Information [file 41467_2023_44384_MOESM1_ESM.pdf]
