# Supplementary material for: Sox9 regulates alternative splicing and pancreatic beta cell function
Source: Nat Commun. 2024 Jan 18;15:588. doi: 10.1038/s41467-023-44384-8 (PMC10796970; doi:10.1038/s41467-023-44384-8)
Supplement: Supplementary file 3 — Description of Additional Supplementary Files [file 41467_2023_44384_MOESM3_ESM.docx]

**Description of Additional Supplementary Files**

**Supplementary Data S1.** **List of differentially expressed genes between Sox9 knockout (KO) islets and control (WT) islets.** Upregulated genes are shown in red and downregulated genes are shown in cyan. A gene-level Wald test (gene_mode=True) between Sox9 knockout (KO) and control (WT) was carried out by aggregating the estimated isoform counts of each gene to their corresponding ensembl gene id in each condition. The raw p-values were used to compute Benjamini-Hochberg-adjusted false discovery rates (q values) and significance was set at 5% FDR.

**Supplementary Data S2**. **Gene ontology analysis for *Biological Processes* of differentially expressed genes between Sox9 knockout (*Ins-Cre;Sox9-/-)* and control islets.** Fisher's Exact test is adopted to measure the gene-enrichment in annotation terms. Fisher's Exact *p*-values are computed by summing probabilities p over defined sets of tables. The Bonferroni in DAVID is the Bonferroni Šidák *p*-value. Benjamini in DAVID requests adjusted *p*-values by using the linear step-up method of Benjamini and Hochberg. FDR in DAVID requests adaptive linear step-up adjusted *p*-values for approximate control of the false discovery rate, as discussed in Benjamini and Hochberg.

**Supplementary Data S3**. **Gene ontology analysis for *Cellular Component* of differentially expressed genes between Sox9 knockout (*Ins-Cre;Sox9-/-)* and control islets.** Fisher's Exact test is adopted to measure the gene-enrichment in annotation terms. Fisher's Exact *p*-values are computed by summing probabilities p over defined sets of tables. The Bonferroni in DAVID is the Bonferroni Šidák *p*-value. Benjamini in DAVID requests adjusted *p*-values by using the linear step-up method of Benjamini and Hochberg. FDR in DAVID requests adaptive linear step-up adjusted *p*-values for approximate control of the false discovery rate, as discussed in Benjamini and Hochberg.

**Supplementary Data S4**. **Gene ontology analysis for *Molecular Function* of differentially expressed genes between Sox9 knockout (*Ins-Cre;Sox9-/-)* and control islets.** Fisher's Exact test is adopted to measure the gene-enrichment in annotation terms. Fisher's Exact *p*-values are computed by summing probabilities p over defined sets of tables. The Bonferroni in DAVID is the Bonferroni Šidák *p*-value. Benjamini in DAVID requests adjusted *p*-values by using the linear step-up method of Benjamini and Hochberg. FDR in DAVID requests adaptive linear step-up adjusted *p*-values for approximate control of the false discovery rate, as discussed in Benjamini and Hochberg.

**Supplementary Data S5**. **KEGG pathway analysis for the differentially expressed genes between Sox9-knockout and control islets.** Fisher's Exact test is adopted to measure the gene-enrichment in annotation terms. Fisher's Exact *p*-values are computed by summing probabilities p over defined sets of tables. The Bonferroni in DAVID is the Bonferroni Šidák *p*-value. Benjamini in DAVID requests adjusted *p*-values by using the linear step-up method of Benjamini and Hochberg. FDR in DAVID requests adaptive linear step-up adjusted *p*-values for approximate control of the false discovery rate, as discussed in Benjamini and Hochberg.

**Supplementary Data S6**. **Enrichr analysis of differentially expressed genes in Sox9-knockout islets.** Terms included in Fig. 2F are highlighted in blue. The p value for the Enrichr is computed from the Fisher Exact test which is a proportion test that assumes a binomial distribution and independence for probability of any gene belonging to any set.

**Supplementary Data S7**. **Alternatively spliced genes in the Sox9-knockout islets.** 315 genes were significantly altered between the knockout and control tissues. A transcript-level Wald test was carried out to capture alternative splicing and isoform switching events between Sox9 knockout (KO) and control (WT), followed by aggregating the resulting p-values to the gene-level (aggregation_column=’ens_gene’). This approach uses the Lancaster method^73^ to compute a single p-value for each gene taking into account the individual p-values of its isoforms. The raw p-values were used to compute Benjamini-Hochberg-adjusted false discovery rates (q values) and significance was set at 5% FDR.

**Supplementary Data S8**. **Genes identified as significantly alternatively splicing using rMATS (as a subset of the top 20 genes identified through Sleuth).** For alternative splicing, p values were generated by sleuth or rMATs as per the software parameters.

**Supplementary Data S9**. **Transcript variants of the murine Srsf5.** Protein coding variants are shown in blue and non-protein coding variants are shown in orange. A transcript-level Wald test was carried out to capture alternative splicing and isoform switching events between Sox9 knockout (KO) and control (WT), followed by aggregating the resulting p-values to the gene-level (aggregation_column=’ens_gene’). The raw p-values were used to compute Benjamini-Hochberg-adjusted false discovery rates (q values) and significance was set at 5% FDR.

**Supplementary Data S10**. **Transcript variants of the murine Srsf6.** The protein-coding variant is shown in blue and non-protein coding variants are shown in orange. A transcript-level Wald test was carried out to capture alternative splicing and isoform switching events between Sox9 knockout (KO) and control (WT), followed by aggregating the resulting p-values to the gene-level (aggregation_column=’ens_gene’). The raw p-values were used to compute Benjamini-Hochberg-adjusted false discovery rates (q values) and significance was set at 5% FDR.

**Supplementary Data S11**. **The top 200 markers in each cluster after analysis of the 10X sequencing data for single cells isolated from iSOX9KO eBCs either treated with or without DOX.** Gene counts were log-normalized, highly variable genes were selected with scanpy’s pp.highly_variable_genes function (flavor=’seurat’) with the default parameters (2410 genes kept) and both total UMI counts and mitochondrial count percentages were regressed out. UMAP dimensionality reduction^78^ was performed on the nearest neighbor graph computed with bbknn^79^ on the PCA-reduced gene expression matrix (50 principal components). Clustering was performed with the Leiden algorithm implemented within scanpy with resolution parameter set to 0.2 and 0.5 to generate coarse and fine-grained resolution cluster labels. Cell types were annotated based on known gene markers and genes identified from a standard differential expression analysis with sc.tl.rank_genes_groups with the default parameters. The resulting gene lists were first filtered based on their p values (<10e-5) and then sorted based on their log-fold change.

**Supplementary Data S12**. **The top 200 genes that are dysregulated in either direction (up or down) upon treatment of iSOX9KO eBCs with DOX.** Gene counts were log-normalized, highly variable genes were selected with scanpy’s pp.highly_variable_genes function (flavor=’seurat’) with the default parameters (2410 genes kept) and both total UMI counts and mitochondrial count percentages were regressed out. UMAP dimensionality reduction^78^ was performed on the nearest neighbor graph computed with bbknn^79^ on the PCA-reduced gene expression matrix (50 principal components). Clustering was performed with the Leiden algorithm implemented within scanpy with resolution parameter set to 0.2 and 0.5 to generate coarse and fine-grained resolution cluster labels. Cell types were annotated based on known gene markers and genes identified from a standard differential expression analysis with sc.tl.rank_genes_groups with the default parameters. The resulting gene lists were first filtered based on their p values (<10e-5) and then sorted based on their log-fold change.
