# Supplementary material for: Sox9 regulates alternative splicing and pancreatic beta cell function
Source: Nat Commun. 2024 Jan 18;15:588. doi: 10.1038/s41467-023-44384-8 (PMC10796970; doi:10.1038/s41467-023-44384-8)
Supplement: Supplementary file 16 — Reporting Summary [file 41467_2023_44384_MOESM16_ESM.pdf]

nature portfolio

Corresponding author(s): Matthias Hebrok

Last updated by author(s): 28/11/23

Reporting Summary

Nature Portfolio wishes to improve the reproducibility of the work that we publish. This form provides structure for consistency and transparency in reporting. For further information on Nature Portfolio policies, see our Editorial Policies and the Editorial Policy Checklist.

Statistics

For all statistical analyses, confirm that the following items are present in the figure legend, table legend, main text, or Methods section.

n/a	Confirmed	
☐	☒	The exact sample size (*n*) for each experimental group/condition, given as a discrete number and unit of measurement
☐	☒	A statement on whether measurements were taken from distinct samples or whether the same sample was measured repeatedly
☐	☒	The statistical test(s) used AND whether they are one- or two-sided<br>*Only common tests should be described solely by name; describe more complex techniques in the Methods section.*
☐	☒	A description of all covariates tested
☐	☒	A description of any assumptions or corrections, such as tests of normality and adjustment for multiple comparisons
☐	☒	A full description of the statistical parameters including central tendency (e.g. means) or other basic estimates (e.g. regression coefficient) AND variation (e.g. standard deviation) or associated estimates of uncertainty (e.g. confidence intervals)
☐	☒	For null hypothesis testing, the test statistic (e.g. *F*, *t*, *r*) with confidence intervals, effect sizes, degrees of freedom and *P* value noted<br>*Give P values as exact values whenever suitable.*
☐	☐	For Bayesian analysis, information on the choice of priors and Markov chain Monte Carlo settings
☐	☐	For hierarchical and complex designs, identification of the appropriate level for tests and full reporting of outcomes
☐	☐	Estimates of effect sizes (e.g. Cohen's *d*, Pearson's *r*), indicating how they were calculated

Our web collection on statistics for biologists contains articles on many of the points above.

Software and code

Policy information about availability of computer code

Data collection	FACSDiva Software 8.0.2 for flow sorting
Data analysis	cutadapt v1.13 for adapting and trimming the raw fastq files, kallisto version 0.46.1 for data analysis, bustools (version 0.40.0) for the transcript compatibility counts generation, rMATS v4.0.2 for the identification of splicing events , STAR v.2.7.3a, FlowJo10.7 for FACS analysis, GraphPad Prism 9.5.1

For manuscripts utilizing custom algorithms or software that are central to the research but not yet described in published literature, software must be made available to editors and reviewers. We strongly encourage code deposition in a community repository (e.g. GitHub). See the Nature Portfolio guidelines for submitting code & software for further information.

Data

Policy information about availability of data

All manuscripts must include a data availability statement. This statement should provide the following information, where applicable:

- Accession codes, unique identifiers, or web links for publicly available datasets
- A description of any restrictions on data availability
- For clinical datasets or third party data, please ensure that the statement adheres to our policy

The data that support the findings of this study are available in a publicly accessible repository and are referenced in the Data Availability statement in the manuscript.

Research involving human participants, their data, or biological material

Policy information about studies with human participants or human data. See also policy information about sex, gender (identity/presentation), and sexual orientation and race, ethnicity and racism.

Reporting on sex and gender	The Human Stem Cell line used in this study was generated at Monash University and we have an MTA in place with Dr. Ed Stanley for use of the cells.
Reporting on race, ethnicity, or other socially relevant groupings	See above.
Population characteristics	See above.
Recruitment	As we did not generate the cells, we were not part of the recruitment to collect donor material.
Ethics oversight	The cells used in our study were generated under the approval of Monash University Human Ethics Committee (2002-225MC).

Note that full information on the approval of the study protocol must also be provided in the manuscript.

Field-specific reporting

Please select the one below that is the best fit for your research. If you are not sure, read the appropriate sections before making your selection.

[x] Life sciences [ ] Behavioural & social sciences [ ] Ecological, evolutionary & environmental sciences

For a reference copy of the document with all sections, see nature.com/documents/nr-reporting-summary-flat.pdf

Life sciences study design

All studies must disclose on these points even when the disclosure is negative.

Sample size	For all the animal experiments, a minimum of three biological replicates were used to measure the variability between animals, driven by the duration of collecting the biological samples. >10 biological samples were used to confirm the phenotype, and subsequent analyses focused on smaller cohorts due to availability of animals of the correct genotype. For the human single cell sequencing analyses, each event was considered a separate replicate for the data analyses. For the stem cell differentiation experiments, multiple differentiations were used for technical replicates as the starting material is the same cell line.
Data exclusions	No data were excluded from the analyses.
Replication	All experiments were successfully repeated at least three times with biological (for animal data) or technical (for stem cell experiments). Stem cell differentiation from the Ins-GFP cell line has been successful >50 times but has not been reported due to space limitations.
Randomization	For animal experiments, genotypes determined the allocation of samples into experimental groups. For human stem cells, the presence or absence of doxycycline treatment determined inclusion in the control or test group.
Blinding	Investigators were blinded to the animal genotypes during data collection and analysis. Investigators were also blinded to the genotypes while conducting sequencing data analyses.

Reporting for specific materials, systems and methods

We require information from authors about some types of materials, experimental systems and methods used in many studies. Here, indicate whether each material, system or method listed is relevant to your study. If you are not sure if a list item applies to your research, read the appropriate section before selecting a response.

Materials & experimental systems

n/a	Involved in the study	
[ ]	[x]	Antibodies
[ ]	[x]	Eukaryotic cell lines
[x]	[ ]	Palaeontology and archaeology
[ ]	[x]	Animals and other organisms
[x]	[ ]	Clinical data
[x]	[ ]	Dual use research of concern
[x]	[ ]	Plants

Methods

n/a	Involved in the study	
[x]	[ ]	ChIP-seq
[ ]	[x]	Flow cytometry
[x]	[ ]	MRI-based neuroimaging

Antibodies

Antibodies used	Mouse monoclonal Anti-Insulin antibody (clone K36AC10, I2018, Sigma)<br>Guinea Pig Polyclonal Anti-Insulin (#A0564, Dako)<br>Rabbit polyclonal Anti-GLUT-2 (#07-1402, Millipore)<br>Mouse monoclonal anti-proinsulin (clone GS9A8, Developmental Studies Hybridoma Bank, University of Iowa)<br>Rabbit policlonal anti-Pdxl (07-696 Millipore)<br>Mouse monoclonal anti-Nkx6.l (F55A10-c, Developmental Studies Hybridoma Bank, University of Iowa)<br>Rabbit policlonal anti-Mafa (IHC-00352, Bethyl labs)<br>Mouse monoclonal anti-Nkx2 (74.5A5-c, Developmental Studies Hybridoma Bank, University of Iowa)<br>Chicken policlonal anti-Green Fluorescent Protein Antibody(GFP-1020, Aves Labs)<br>Rabbit policlonal RFP Antibody Pre-adsorbed (600-401-379, Rockland)<br>Rabbit policlonal anti-SOX9 (Prestige HPA001758, Sigma)<br>Goat anti-Mouse IgG (H+L) Highly Cross-Adsorbed Secondary Antibody, Alexa Fluor™ 488 (A-11029, Invitrogen)<br>Goat anti-Rabbit IgG (H+L) Highly Cross-Adsorbed Secondary Antibody, Alexa Fluor™ 488 (A11034, Invitrogen)<br>Goat anti-Guinea Pig IgG (H+L) Highly Cross-Adsorbed Secondary Antibody, Alexa Fluor™ 488 (A-11073, Invitrogen)<br>Goat anti-Rabbit IgG (H+L) Cross-Adsorbed Secondary Antibody, Alexa Fluor™ 555 (A21428, Invitrogen)<br>Goat anti-Guinea Pig IgG (H+L) Highly Cross-Adsorbed Secondary Antibody, Alexa Fluor™ 555 (A21422, Invitrogen)<br>Goat anti-Mouse IgG (H+L) Cross-Adsorbed Secondary Antibody, Alexa Fluor™ 555 (A21435, Invitrogen)<br>Goat anti-Guinea Pig IgG (H+L) Highly Cross-Adsorbed Secondary Antibody, Alexa Fluor™ 633 (A21105, Invitrogen)<br>Biotin-SP (long spacer) AffiniPure Goat Anti-Rabbit IgG (H+L) (111-065-003, Jackson ImmunoResearch)<br>Goat Anti-Guinea Pig IgG Antibody (H+L), Biotinylated (BA-7000, Vector Labs)<br>Human anti- PAX6-Alexa647, 1:50 (#562249, BD Bioscience)<br>Islet-1-PE, 1:50 (#562547, BD Bioscience)<br>NKX6.1-Alexa647, 1:50 (#563338, BD Bioscience)<br>NKX2.2-PE, 1:50 (#564730, BD Bioscience)<br>ChromograninA-PE, 1:50 (#564563, BD Bioscience)<br>NeuriD1-Alexa647, 1:50 (#563566, BD Bioscience)<br>Glucagon-Alexa647, 1:2000 (#G2654, Sigma, mouse antibody conjugated in-house)<br>C-peptide-488, 1:200 (C-PEP-01, Chemicon, mouse antibody conjugated in-house)<br>Human SOX17-Alexa488, 1:50 (#P7-969, BD Bioscience)<br>Human FOXA2-PE, 1:50 (#N17-280, BD Bioscience)
Validation	No validation statements for commercially available antibodies included here as all primary antibodies are validated by the vendors.

Eukaryotic cell lines

Policy information about cell lines and Sex and Gender in Research

Cell line source(s)	The INS-GFP MEL-1 line was kindly provided by Ed Stanley (Micallef et al., 2012) and serves as parental line from which the iSOX9KO line was generated.
Authentication	We have generated beta cells from this cell line, and the expression of GFP in these cells validates the insertion of GFP into the INSULIN locus.
Mycoplasma contamination	We routinely test our cells for mycoplasma infections, and the cells used in this study were negative for mycoplasma.
Commonly misidentified lines (See ICLAC register)	No commonly misidentified cell lines were used in this study.

Animals and other research organisms

Policy information about studies involving animals; ARRIVE guidelines recommended for reporting animal research, and Sex and Gender in Research

Laboratory animals	Mice were maintained at 21–23°C with 30–70% humidity and a 12h dark/light cycle in a pathogen-free facility at UCSF. Mice were provided with food and water ad libitum and were housed in autoclaved individually ventilated caging in groups of up to 5 mice per cage. Mice were bred and grown in house and maintained in the same facility included the following strains: Sox9fl/fl (C57 background), Ins-Cre (C57 background), MIP-CreER (mixed background) and mTmG (C57 background) mice were used in this study. For the Cre positive animals, males were used for the physiological analyses. For all animals, physiological tests were carried out in animals 8 weeks and older.
Wild animals	No wild animals were used in this study.
Reporting on sex	Physiological analyses were carried out on male mice unless otherwise noted, as we observe gender differences in our animal

	colonies (for all the Cre-Sox9 knockout cohort, with a small n number, we do not see a phenotype in the females, and have restricted our analysis to only the males). Ex vivo analysis were carried in both males and females islets since we did not see any difference between our groupes.
Field-collected samples	No field collected samples were used in this study.
Ethics oversight	Ethical oversight was provided by the UCSF IACUC committee.

Note that full information on the approval of the study protocol must also be provided in the manuscript.

Flow Cytometry

Plots

Confirm that:

- [x] The axis labels state the marker and fluorochrome used (e.g. CD4-FITC).
- [x] The axis scales are clearly visible. Include numbers along axes only for bottom left plot of group (a 'group' is an analysis of identical markers).
- [x] All plots are contour plots with outliers or pseudocolor plots.
- [x] A numerical value for number of cells or percentage (with statistics) is provided.

Methodology

Sample preparation	Clusters at indicated stages were dissociated, fixed, permeabilized and stained for various intracellular markers for analysis
Instrument	LSRFortessa X20 Dual
Software	FACSDiva Software 8.0.2 and FlowJo10.7
Cell population abundance	Sorting efficiency ranges from 40 to 70%. This figure is calculated on the basis of GFP-expressing beta cells.
Gating strategy	Cells were first gated for live cells in FSC-A (x-axis) versus SSC-A (y-axis). We identify single cells in SSC-W versus FSC-H. We then gate for Beta cells in SSC-W versus GFP-A

- [x] Tick this box to confirm that a figure exemplifying the gating strategy is provided in the Supplementary Information.
